# Detailed Simulations of Cell Biology with Smoldyn 2.1

**DOI:** 10.1371/journal.pcbi.1000705

**Published:** 2010-03-12

**Authors:** Steven S. Andrews, Nathan J. Addy, Roger Brent, Adam P. Arkin

**Affiliations:** 1 Physical Biosciences Division, Lawrence Berkeley National Laboratory, Berkeley, California, United States of America; 2 National Centre for Biological Sciences, Tata Institute for Fundamental Research, Bangalore, India; 3 Molecular Sciences Institute, Berkeley, California, United States of America; 4 Department of Bioengineering, and Howard Hughes Medical Institute, University of California, Berkeley, California, United States of America; 5 Virtual Institute for Microbial Stress and Survival, http://vimss.lbl.gov; University of Washington, United States of America

## Abstract

Most cellular processes depend on intracellular locations and random collisions of individual protein molecules. To model these processes, we developed algorithms to simulate the diffusion, membrane interactions, and reactions of individual molecules, and implemented these in the Smoldyn program. Compared to the popular MCell and ChemCell simulators, we found that Smoldyn was in many cases more accurate, more computationally efficient, and easier to use. Using Smoldyn, we modeled pheromone response system signaling among yeast cells of opposite mating type. This model showed that secreted Bar1 protease might help a cell identify the fittest mating partner by sharpening the pheromone concentration gradient. This model involved about 200,000 protein molecules, about 7000 cubic microns of volume, and about 75 minutes of simulated time; it took about 10 hours to run. Over the next several years, as faster computers become available, Smoldyn will allow researchers to model and explore systems the size of entire bacterial and smaller eukaryotic cells.

## Author Summary

We developed a general-purpose biochemical simulation program, called Smoldyn. It represents proteins and other molecules of interest with point-like particles that diffuse, interact with surfaces, and react, all in continuous space. This high level of detail allows users to investigate spatial organization within cells and natural stochastic variability. Although similar to the MCell and ChemCell programs, Smoldyn is more accurate and runs faster. Smoldyn also supports many unique features, such as commands that a “virtual experimenter” can execute during simulations and automatic reaction network expansion for simulating protein complexes. We illustrate Smoldyn's capabilities with a model of signaling between yeast cells of opposite mating type. It investigates the role of the secreted protease Bar1, which inactivates mating pheromone. Intuitively, it might seem that inactivating most of the pheromone would make a cell less able to detect the local pheromone concentration gradient. In contrast, we found that Bar1 secretion improves pheromone gradient detectability: the local gradient is sharpened because pheromone is progressively inactivated as it diffuses through a cloud of Bar1. This result helps interpret experiments that showed that Bar1 secretion helped cells distinguish between potential mates, and suggests that Bar1 helps yeast cells identify the fittest mating partners.

## Introduction

One hurdle to the computational modeling of cellular systems is the lack of adequate tools. If one assumes that molecules inside cells are well-mixed, and that they behave deterministically, then one can model the chemical reactions that cells use to operate with differential equations (recently reviewed by Alves and coworkers [1]). However, these assumptions are frequently inadequate. Firstly, most cellular processes depend at least to some extent on intracellular spatial organization. For example, cell signaling systems transmit signals across significant distances within subcellular compartments and across intracellular membranes, such as the nuclear envelope. Also, cell division systems segregate one cell into two and regulate the partition of molecular components. Secondly, many cellular outputs exhibit substantial random variation [2], which must arise from random differences in molecular collisions. Examples range from the random switching of swimming *Escherichia coli* bacteria between so-called running and tumbling states [3] to cell-to-cell variation in the operation of cell signaling systems [4],[5]. More generally, stochastic behavior is likely to affect the outcomes of essentially all cellular processes. Representation of this complexity requires algorithms and programs that model cellular processes with spatial accuracy [6], and that model the chemical reactions by which they operate with stochastic detail [7].

Computational biologists have pursued four main approaches to simulating biochemical systems with spatial and stochastic detail. These differ in how they represent space, time, and molecules (Table 1), which in turn affects the classes of biological systems that they can simulate appropriately. (*i*) The *spatial Gillespie method* [8] is based on Gillespie's stochastic simulation algorithms [9]. It divides the simulation volume into a coarse lattice of subvolumes, each of which contains many molecules of interest. This method can be computationally efficient because it tracks the total number of individual classes of molecules per subvolume, rather than individual molecules [10]. However, the lattice structure it uses to divide space into subvolumes does not work well for realistic membrane shapes, which require special treatment [11]. (*ii*) The *microscopic lattice method* subdivides space into a much finer lattice, so that each volume can contain zero or one molecule. In this method, molecules diffuse by hopping between sites and can react with molecules in neighboring sites. It naturally lends itself to studies of oligomerization and complex formation [12], and of the effects of macromolecular crowding on reactions [13]. It has not found wide use for studying cell-sized processes due to the facts that it has high computational demands and specific lattice structures affect simulated reaction rates differently [14], although recent techniques may circumvent these challenges [15]. (*iii*) *Particle-based methods*, the primary focus of this article, are the most widely used spatial stochastic methods [7]. These represent individual molecules with point-like particles that diffuse in continuous space over fixed time steps; molecules can react when they collide. The fact that these models use continuous space makes realistic membrane geometries relatively easy to represent [16], avoids lattice-based artifacts, and offers high spatial resolution. The need to track individual molecules, however, imposes high computational demands, so particle-based methods are about a factor of two slower than spatial Gillespie methods [10]. Finally, (*iv*) *Green's function reaction dynamics* (*GFRD*) *methods* [17] enable especially accurate particle-based simulation. GFRD methods step the simulation from the exact time of one individual reaction to the exact time of the next. This makes these methods ideal for systems that can have long delays between individual reactions, but very computationally intensive for most cellular processes [10].

**Table 1:** Biochemical simulation methods that account for spatial and stochastic detail.

Simulation method	Simulation programs	Space representation	Time treatment	Molecule representation
Spatial Gillespie	MesoRD [68]	coarse lattice	event-based	populations
	SmartCell [69]			
	GMP [70]		a	
Fine lattice	GridCell [71]	fine lattice	fixed steps	individuals
	Spatiocyte [15]			
Particle-based	ChemCell [72]	continuous	fixed steps	individuals
	MCell [20]		b	
	Cell++ [73]	c		c
	Smoldyn (this work)			
GFRD	E-Cell^d^	continuous	event-based	individuals

a GMP is event-based for reactions and uses fixed time steps for diffusion.

b MCell uses an event-based scheduling system in which short steps are used for fast processes and long steps for slow processes.

c Cell++ represents small molecules, such as metabolites, as concentrations on a coarse lattice and large molecules, such as enzymes, as individual particles in continuous space.

d GFRD is in process of being added to E-Cell.

The dominant particle based simulators are ChemCell [18], MCell [19],[20], and Smoldyn [21],[22] (see also Table 2). These programs have many common features, but differ in other features and in the quantitative accuracy of their simulations. Of the three, ChemCell has the fewest features, but is particularly easy to use and is the only simulator that supports both spatial and non-spatial simulations. MCell, the oldest program [23], has been used the most, produces the highest quality graphics [16], and has a number of features that make it particularly well suited to simulating cellular processes involved in synaptic transmission [19] (for example, using MCell, it is easy to release agonist and antagonist ligands onto a cell using pulse trains). Smoldyn is a relative newcomer, but yields the most accurate results and runs the fastest (see Text S1). Smoldyn also has a number of attributes, listed in Table 2 and below, which make it well suited to modeling a wide range of cellular processes.

**Table 2:** Comparison of current particle-based simulators.

Feature	ChemCell	MCell3	Smoldyn 2.1
Simulation methods	ODE, Gillespie, particle	particle	particle
Time steps	fixed	adaptive	fixed
System dimensionality	3	3	1, 2, 3
System boundaries	reflective, periodic	reflective, absorbing, transparent	reflective, absorbing, periodic, transparent
Geometry primitives	triangles, spheres, boxes, planes, cylinders	triangles	triangles, rectangles, spheres, cylinders, hemispheres, disks
Surface molecule states	transmembrane	integral states: top-front, top-back	integral, peripheral states: up, down, front, back
Accuracy of diffusion	volume: exact	volume: exact	volume: exact
	surface: approx	surface: approx.	surface: approx.
Accuracy of reactions in solution	order 0: none	order 0: none	order 0: exact
	order 1: exact	order 1: exact	order 1: exact
	order 2: approx.	order 2: approx.	order 2: exact
Accuracy of reactions on surfaces	order 0: none	order 0: none	order 0: exact
	order 1: exact	order 1: exact	order 1: exact
	order 2: not quantitative	order 2: approx.	order 2: not quantitative
Dissociation reaction product placement	at reactants, not quantitative	stochastic, for microscopic reversibility	fixed separation, for accurate reaction rates
User can fix molecular concentrations	no	near surfaces	on surfaces, in compartments
Location-specific reactions	no	surfaces	surfaces, compartments
Surface interactions	adsorb: not quantitative	adsorb: not quantitative	adsorb: exact
	desorb: exact	desorb: exact	desorb: exact
	permeable: not quantitative	permeable: not quantitative	permeable: exact
Parallel processing	MPI	MPI	POSIX threads
Graphics	post-run with pizza.py	post-run with DReAMM	during simulation
Source code	open, GPL license	closed	open, GPL license
Benchmark run time	99 s	120 s	47 s
Computer systems	Mac, Linux	Mac, Linux, Windows	Mac, Linux, Windows

Please see Text S1 for details and other comparisons.

**Figure 1:**
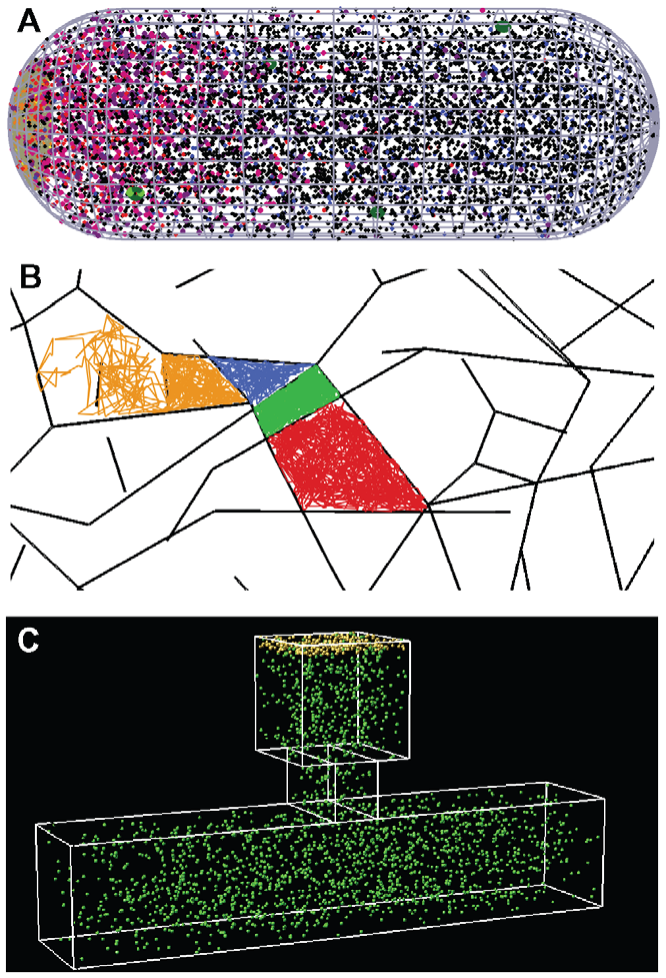
Example Smoldyn models. (A) Model of *Escherichia coli* chemotaxis, adapted from work by Lipkow and Odde [29]. Receptors are clustered at the left cell pole, unphosphorylated CheY messenger proteins are shown in black and phosphorylated CheY are in red. This simulation showed that the combination of a spatially segregated kinase-phosphatase system and phosphorylation-dependent diffusion coefficients can create stable protein gradients. (B) Simulated track of a diffusing lipid molecule, which is on a membrane that is divided into “corrals” by underlying actin filaments, shown with black lines. The track is shown in red, then green, blue, and yellow to illustrate that lipids tend to fully explore individual corrals before hopping to adjacent corrals. Actin data are from Morone et al. [74]. (C) Model of a dendritic spine, showing GFP-tagged calcium calmodulin dependent kinase II proteins (CaMKII) in green and molecules of the postsynaptic density at the spine tip in yellow, adapted from work by Khan et al. [26]. The authors used these simulations to analyze fluorescence microscopy data and to investigate CaMKII sequestration upon synaptic excitation.

This article focuses on the latest version of Smoldyn, Smoldyn 2.1. Smoldyn 1.0 embodied several algorithms that were based on Smoluchowski reaction dynamics [21]. It and subsequent versions were used to investigate a spatial version of the classic Lotka-Volterra chemical oscillator [24], diffusion on hair cell membranes [25], protein sequestration in dendritic spines [26], diffusion in obstructed spaces, and intracellular signaling in *E. coli* chemotaxis [27]–[29] (Figure 1). Smoldyn 2.1 preserves the original focuses on accuracy and efficiency but offers significantly improved functionality. In particular, it can represent realistic membrane geometries, simulate diffusion of membrane-bound molecules, and accurately simulate a wide variety of molecule-membrane interactions [30]. To make it as general a simulator as possible, Smoldyn 2.1 also supports spatial compartments, rule-based reaction network generation [31],[32], molecules with excluded volume, conformational spread interactions, and over fifty run-time commands for system manipulation and observation. We anticipate that Smoldyn will be particularly useful for (*i*) investigating cellular systems, such as signaling, division, and metabolic systems, (*ii*) studying basic biophysical phenomena, such as the effects of macromolecular crowding on molecular diffusion, and (*iii*) helping to quantify microscopy data [33], such as diffusion rates investigated by FRAP (fluorescence recovery after photobleaching, which is based on the time it takes fresh fluorophores to diffuse into the bleached volume).

## Results

### Design philosophy

The algorithms that Smoldyn uses, and the program's name, derive from a biophysical description of space and chemical reactions that von Smoluchowski defined in 1917 [34]. In his description, each molecule of interest moves by mathematically ideal Brownian motion [35], which implicitly arises from its collisions with solvent molecules and other non-reacting molecules. Bimolecular chemical reactions occur when two reactants diffuse to within a distance called the encounter radius [36], or binding radius [21]. With the Smoldyn algorithms, all simulated dynamics approach those of the Smoluchowski model (within computer round-off error) as the simulation time step is reduced towards zero.

### Workflow

Smoldyn reads a configuration file that describes the system or cellular process under study. This file lists the system dimensionality, initial numbers of molecules, membrane locations, chemical reactions, and the rules for molecule-surface interactions. It also contains directives for a virtual experimenter, a software agent under the direction of the human researcher, which can measure and manipulate the system during its simulation. For example, at any given point during the simulation run, the virtual experimenter can count the number of particular molecules or add a new surface to represent intrusion of a membrane vesicle into the simulated space. After calculating simulation parameters, Smoldyn performs the simulation with fixed-length time steps, typically set by the researcher to be shorter than characteristic reaction or diffusion timescales (0.1 ms time steps often work well [27]–[29], but see [22] for a more thorough discussion). At each time step, Smoldyn diffuses simulated molecules, performs chemical reactions, processes molecule-membrane interactions, and outputs quantitative data to one or more text files. Smoldyn can display the simulated system to a graphics window as it is computed, or it can work in a text-only mode for more efficient operation.

### Molecules and surfaces

Smoldyn represents *molecules* and *surfaces*. Molecules can be in free solution, such as cytoplasmic proteins, or bound to surfaces, such as ion channels or peripheral membrane proteins. Surface-bound molecules can bind to the front or back of the surface, or can be transmembrane with an “up” or “down” orientation. The latter two states can differentiate the two orientations of transmembrane proteins, such as whether the ligand-binding portion of a receptor faces the intra- or extracellular medium.

Surfaces, which might represent biological membranes or the sides of a reaction vessel, are modeled as being infinitely thin and locally smooth. Each surface is composed of *panels*. Panels can be rectangular, triangular, spherical, hemispherical, cylindrical, or disk-shaped. Of course, one only needs triangles to model arbitrarily complex surfaces [37] and for that reason triangle-based meshes are widely used for experimentally-derived membrane geometries (reviewed by O'Rourke [38]). However, the other panel shape options can simplify membrane definitions and improve computational efficiency. For example, Smoldyn can represent a nuclear membrane as a sphere rather than as an icosahedron. Two utility programs, distributed with Smoldyn, simplify surface definitions: one (wrl2smol) converts triangle data from the Virtual Reality Modeling Language format, which is widely used for microscopy, to Smoldyn format, and the other (SmolCrowd) generates fields of random non-overlapping circles or spheres for investigating macromolecular crowding.

### Algorithms

**Figure 2:**
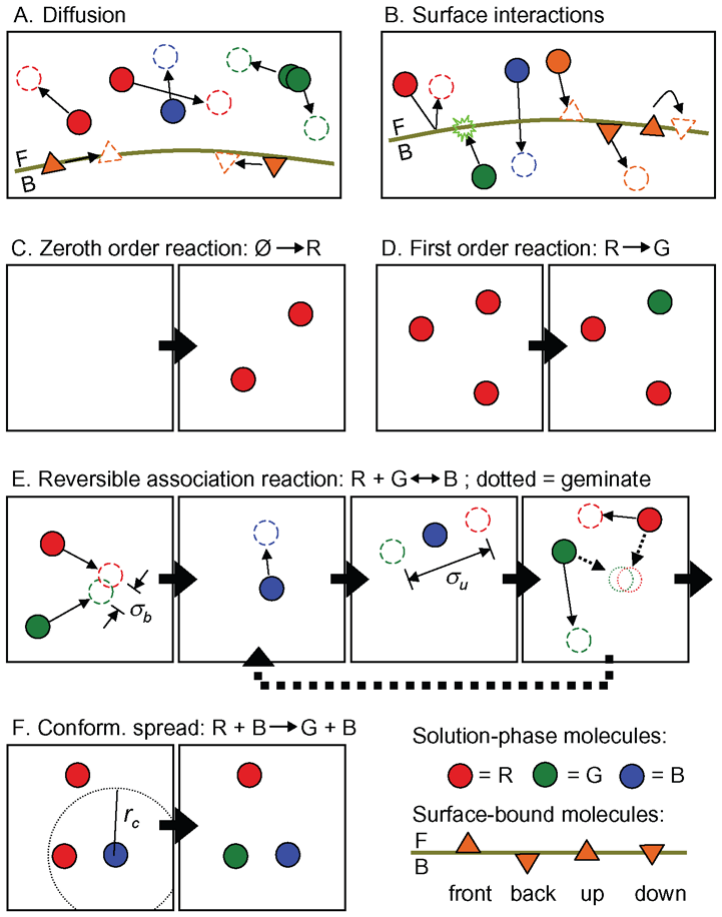
Algorithms used in Smoldyn. The bottom-right panel is a key. The front and back sides of surfaces are noted with ‘F’ and ‘B’, respectively. (A) Diffusion for solution and surface-bound molecules; note that there is no excluded volume. (B) From left to right, interactions between molecules and surfaces are: reflection, absorption, transmission, adsorption, desorption, and surface-state conversion. (C) Zeroth order chemical reaction. (D) First order chemical reaction. (E) In these sequential association and dissociation reactions, *α_b_* is the binding radius and *α_u_* is the unbinding radius. The last frame shows two possible scenarios, which are the geminate recombination of the dissociation products (dashed arrows) or diffusion of these products away from each other (solid arrows). (F) Conformational spread reaction with interaction distance *r_c_*.

We summarize the core algorithms here and in Figure 2. For more detail, see [21],[22],[30] or the Smoldyn user's manual (supplied with the program).

(*i*) To simulate diffusion, Smoldyn moves each molecule at each time step using Gaussian-distributed random displacements along each *x*, *y*, and *z* coordinate [21]. If the molecule was surface-bound, Smoldyn then deposits it back upon the surface along the local normal vector (an exact method for planar surfaces because orthogonal projections of 3-dimensional Gaussian probability densities are 2-dimensional Gaussian probability densities; for this to be accurate for curved surfaces, the radius of curvature needs to be much larger than the length of the average diffusive step). Smoldyn can also simulate molecular drift, for example arising from a flow of solvent molecules, and anisotropic diffusion.

(*ii*) To simulate interactions between solution-phase molecules and membranes, Smoldyn simulates impermeable membranes with ballistic reflections [21], partially permeable and adsorbing membranes [39] using interaction probabilities that are exact at steady-state and very accurate away from steady-state [30], and periodic (or toroidal) boundaries with so-called jump surfaces, with which molecules that diffuse out of one side of the system immediately diffuse into the opposite side. These jump surfaces can also be used to add holes to otherwise impermeable surfaces. In addition, “unbounded-emitter” surfaces cause molecular concentrations to equal those that would be observed if the system were unbounded, by absorbing molecules with probabilities based on the emitter positions [30].

(*iii*) To handle transitions from surface-bound states, Smoldyn assigns these reactions a probability computed as described below for first order reactions. Smoldyn then displaces desorbed molecules away from the surface using probability densities that account for diffusion that occurs between the time of desorption and the end of the time step in which desorption occurred (the density is an error function if desorption is reversible and combines a Gaussian and an error function if desorption is irreversible [30]).

(*iv*) To handle reaction products without the respective reactants, Smoldyn simulates “zeroth” order chemical reactions. This allows Smoldyn to represent, for example, protein expression, as the *de novo* appearance of protein in the reaction volume. Smoldyn adds a Poisson-distributed random number of product molecules to random locations in the simulation volume, or within a smaller compartment (such as a cell nucleus), at each time step [21].

(*v*) To handle first order reactions, such as protein conformational changes, Smoldyn performs each reaction with probability 1–exp(–*k*Δ*t*), where *k* is the reaction rate constant and Δ*t* is the time step, at each time step. Smoldyn uses an expanded version of this equation if a single reactant can undergo any of several reactions [19],[21].

(*vi*) Smoldyn performs second order reactions, which have the form A + B → C, when two reactants diffuse closer together than their “binding radius.” Smoldyn computes these distances from reactant diffusion coefficients, reaction rate constants, and the simulation time step [21], with results that are typically similar to the sums of the physical radii of the reactants (*e.g.* reactants with 10 µm^2^s^−1^ diffusion coefficients and a 10^6^ M^−1^s^−1^ reaction rate constant have a binding radius of 3.4 nm when a 0.1 ms time step is used). Tournier *et al.* [40] showed that this method is indistinguishable from a more accurate one in which the reaction probability varies as a function of the inter-molecular separation. Although Erban and Chapman recently extended our method to include a fixed reaction probability, so that binding radii would be larger [41], Smoldyn does not support their extension because it is less computationally efficient and it does not account for reversible reactions. Neither Smoldyn nor Smoluchowski theory directly simulate reactions with orders that are greater than two or non-integer, but rather decompose these into individual bimolecular reactions.

(*vii*) Smoldyn typically deposits reaction products at the reaction location. However, it places them elsewhere in three important cases. (*a*) If two reaction products can react with each other, Smoldyn separates them by a distance called the *unbinding radius* [21] (typically similar to the binding radius for these molecules), which produces accurate reaction rates by reducing the probability of product recombination [42]. (*b*) If the reaction simulates *conformational spread*, in which allosteric changes in protein activity are transmitted by direct contact between neighboring proteins [43], then Smoldyn places the products at the same locations previously occupied by the reactants. For conformational spread, the typical reaction notation is A + B → A' + B, with the interpretation that B converts A to A' through direct contact. Smoldyn simulates conformational spread reactions with first order reaction rates if the reactants are within a pre-defined interaction distance. (*c*) If the reaction is used to give molecules excluded volume, then Smoldyn separates the products by a distance that is slightly larger than the binding radius and places them on the line that connected the reactants. While this method is not as accurate as ballistic reflection methods, it is still useful; for example, it can assure that molecules cannot pass each other in pores and it can be used to separate molecules by realistic distances.

(*viii*) So that the user does not need to enumerate the “combinatorial explosion” [44] of individual species and reactions that result when proteins form multimeric complexes or are post-translationally modified (*e.g.* phosphorylation), Smoldyn generates species and reactions automatically from lists of interaction rules. These rules include molecule binding sites, modification sites, and allosteric interactions. Smoldyn performs this reaction network expansion as new species and reactions arise in a simulation [45] using the libmoleculizer software module [32] (derived from Moleculizer [31]).

Taken together, this set of algorithms allows Smoldyn to represent most biochemical processes that take place among proteins and small molecules, in 1, 2, or 3 dimensional space and on surfaces and membranes. For example, this capability will bring most cell signaling systems within reach of particle-based modeling. Currently absent, however, from Smoldyn and nearly all comparable simulators are algorithms that specifically address moving or distorting membranes, and the dynamics of biopolymers (including microtubules, actin filaments, and most conspicuously, DNA).

### Accuracy and efficiency

**Figure 3:**
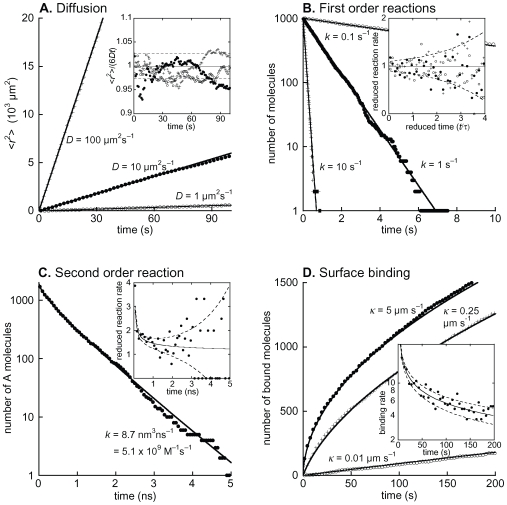
Tests of Smoldyn accuracy. Each panel presents simulation results with points and theoretical results for the same parameters with solid lines. Where present, different shape points represent simulation data for different rates. Inset panels present fluctuations of the same simulation data shown in the main panels, with parameters that are reduced so as to highlight the fluctuations and provide meaningful comparisons between data sets. Here, solid lines represent theoretical expectation values and dashed lines are drawn one standard deviation, analytically calculated from theory, above and below the expectation values. Configuration files and analytic calculations are included with Text S2. (A) Mean square displacements of three populations of freely diffusing molecules that have the listed diffusion coefficients. (B) First order decay reactions (A → Ø) of three populations of molecules that have the listed reaction rate constants. The reduced reaction rate is the reaction rate per molecule per unit of reduced time. (C) Bimolecular association and decay reaction between A and B molecules (A + B → Ø), using the same parameters that are presented in Figure 7 of Andrews and Bray [21]. The reduced reaction rate is the reaction rate per A molecule. (D) Adsorption of molecules that are uniformly distributed initially to planar surfaces with the listed adsorption coefficients. The inset panel shows fluctuations in the *κ* = 5 µm s^−1^ simulation data.

Figure 3 shows that simulation data for several of the core Smoldyn algorithms agree well with analytical theory for wide ranges of rate constants, and that this holds for both cumulative results and fluctuations. Using Pearson's χ^2^ test, we found no statistically significant differences between data and theory (see Text S2). These agreements, along with additional tests presented in Text S1, the Smoldyn user's manual and theoretical work in the algorithm derivations [21],[30], show that individual Smoldyn algorithms are quite accurate both at and away from steady state. In addition, Smoldyn simulations that combine multiple algorithms become exact as time steps are reduced towards zero. On the other hand, Smoldyn simulations with multiple processes and finite time steps are necessarily approximate, in part because individual simulated molecules can only take part in a single reaction or adsorption events during single time steps.

Smoldyn simulation run times scale linearly with the number of simulated molecules (Text S3). However, the number of chemical reactions minimally affects Smoldyn run times because the Smoldyn algorithms iterate over molecules rather than possible reactions. In contrast, spatial Gillespie methods scale linearly with the number of reactions [10], or logarithmically if they use the Gibson-Bruck algorithm [46].

We compared the run times for ChemCell, MCell, and Smoldyn using identical models of a Michaelis-Menten reaction. These models comprised 10,000 molecules (10% enzyme, 90% substrate initially) and ran for 10 s of simulated time in 1 ms time steps. As Table 2 shows, Smoldyn took 47 s to run this test (on a MacBook Pro laptop with a 2.33 GHz Intel Core 2 Duo processor and OS 10.4.11, and with single-threaded operation), which was more than a factor of 2 faster than either ChemCell or MCell. All simulated results agreed well with mass action theory (Text S1). To give a sense of scale, there are 11,200 molecules in Figure 1A [29], about 40,000 protein molecules that regulate chemotaxis in a single *E. coli* [47], and about 40,000 proteins in the core pheromone response system in a single *Saccharomyces cerevisiae*. This comparison is likely to be representative of typical simulations because bimolecular reaction simulation comprises most of the run time for Smoldyn, and likely for the other simulators as well. Smoldyn also supports multiple program threads although these do not provide substantial speed improvements at present.

### Example: the effect of Bar1 on yeast signaling

**Figure 4:**
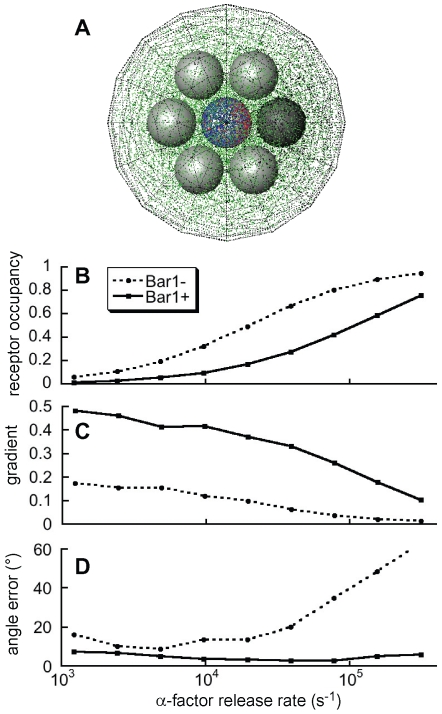
A model of the effect of the Bar1 protease on yeast signaling. (A) A snapshot of the system shown at steady state and with a target cell release of about 4×10^4^ α-factor molecules per second. It is surrounded by a triangulated spherical boundary which absorbs molecules with Smoldyn's “unbounded-emitter” method. The central sphere is a *MAT* **a** cell, light grey spheres are challenger *MAT*α cells, and the dark grey sphere on the right is the target *MAT*α cell. Blue dots are unliganded GPCRs, red are GPCR-α complexes, green are Bar1 molecules, and black are α-factor molecules. Model details are presented in Text S4. (B) Average receptor occupancy for Bar1^+^ and Bar1^–^ cells (see legend). These data fit well to Hill functions with unit cooperativity but with a 5-fold difference in their EC_50_s. (C) Average gradient of GPCR-α complexes across the surface of the *MAT* **a** cell. It is scaled so that 0 represents no gradient and 1 represents the maximum possible gradient. (D) Angle difference between vectors that point from the *MAT* **a** cell center to (*i*) the average position of the GPCR-α complexes and to (*ii*) the target cell center, averaged over all time points. This figure shows that although Bar1 decreases α-factor binding to the *MAT* **a** cell, it increases the *MAT* **a** cell's ability to locate the target *MAT*α cell.

We used Smoldyn's capabilities to explore a simple model. When haploid *Saccharomyces cerevisiae* of opposite mating types *(MAT* **a** and *MAT*α) are in proximity, they can mate and form a diploid [48]. *MAT* **a** cells detect a pheromone (α-factor), secreted by *MAT*α cells, and use the concentration and gradient of the pheromone to grow toward and discriminate among potential *MAT*α partners [49],[50]. Exposure to pheromone also increases the rate at which *MAT* **a** cells secrete a pheromone-degrading protease, Bar1 [51],[52]. Barkai *et al.* showed that a uniform concentration of Bar1 would attenuate α-factor signals coming from distant *MAT*α cells more than from close cells, thus helping *MAT* **a** cells locate the closest potential mating partners [53]. However, this result does not apply to low densities of *MAT* **a** cells. Here, the Bar1 concentration should be highest near *MAT* **a** cells, both because Bar1 binds to cell walls [54] and because Bar1 dissipates as it diffuses away. This is precisely the experimental setup in partner discrimination assays, such as that shown in Figure 4A. In these assays, *MAT* **a** cells are surrounded by *MAT*α cells that secrete different amounts of α-factor. Jackson and Hartwell found that *MAT* **a** cells usually choose the strongest pheromone emitter, and make this choice most accurately when the *MAT* **a** cells express Bar1 [50],[55]. We modeled this experiment with Smoldyn.

Figure 4A shows a snapshot of the Smoldyn simulation. There are two kinds of *MAT*α cells: one cell secretes a normal level of α-factor (the “target” cell) and other cells secrete 5% of the normal α-factor level due to mutations in *mfα1 MFα2* [50]. These cells surround a central *MAT* **a** cell. Onto the surface of the *MAT* **a** cell we introduced 6622 stationary Ste2 receptor molecules [56] which bound α-factor according to experimentally measured rates [57]. In some simulations, the *MAT* **a** cell secreted Bar1. We chose the Bar1 secretion rate, and its catalytic proteolysis reaction rate, so that Bar1 would increase the receptor occupancy EC_50_ by about a factor of 5 (Figure 4B), which is a conservative estimate for the experimental shift [57]. The simulations shown here did not permit Bar1 to bind to cell membranes for simplicity, but agree well with others (not shown) that permitted binding. Finally, we estimated diffusion coefficients with the Stokes-Einstein equation, using the assumption that the extracellular viscosity is similar to that of mammalian cytoplasms [58]. (See Text S4 for further details.)

We simulated systems at each of nine α-factor secretion rates. During the first 100 s of each secretion rate, the systems equilibrated to a nearly steady state, which we assessed using time-dependent concentrations and concentration gradients of all simulated molecular species. Every 2 s for the next 400 s, Smoldyn recorded the number and the mean position of receptor molecules bound to α-factor. We defined the vector that pointed from the *MAT* **a** cell center to one of these mean positions, which we label **r**, to be the “position signal” that the *MAT* **a** cell received. Each vector represented the cell's instantaneous measurement of the local 3-dimensional α-factor gradient. Next, we defined the “GPCR-α gradient” as the component of **r** that points towards the target *MAT*α target cell, divided by the radius of the *MAT* **a** cell. This created a simple metric that could range from –1 to 1; it is 0 if α-factor binds randomly to receptors, and is 1 if α-factor only binds the receptors closest to the target *MAT*α cell.

Figure 4C shows GPCR-α gradient values, averaged over all time points of each α-factor secretion rate. It shows that Bar1 substantially increased the measured gradient of receptor-bound pheromone over a wide range of α-factor secretion rates. This occurred because Bar1 degraded α-factor molecules as they diffused past the *MAT* **a** cell, which steepened the local α-factor concentration gradient. Figure 4D shows a related but likely more physiologically relevant quantity: it shows the average absolute angle between the **r** vectors and the direction to the target cell. Again, the average is over all time points of each α-factor secretion rate. This figure shows that Bar1 decreased the *MAT* **a** cell's angular measurement error, again over a very wide range of α-factor secretion rates. We believe that this improvement arose from the steeper concentration gradients. These effects are consistent with Jackson and Hartwell's finding that *MAT* **a** cells choose the *MAT*α cell that produces the highest level of pheromone from among potential mating partners [50]. In further simulations, we surrounded a *MAT* **a** cell with 3 target and 3 low-secreting *MAT*α cells and found that Bar1 had the same discrimination enhancing effect. Again, this paralleled experimental results [50].

Many features of Smoldyn facilitated the above investigation. The simulations used nearly diffusion-limited reaction rates for the Bar1 protease reaction, which Smoldyn handles accurately; they tracked up to 190,000 molecules at a time, which required high computational efficiency; and they used system boundaries that absorbed molecules so that the distribution of molecules in the bounded system roughly matched the distribution if space extended indefinitely [30]. In addition, Smoldyn's real-time graphics helped us design the simulation and the ability to use spherical cells simplified model building and accelerated simulation run time. We could have used rule-based modeling to automatically generate the receptor-ligand complex species and its reactions, but declared them explicitly instead to make the model simpler.

## Discussion

Many aspects of the biochemical reactions that animate cellular processes are inherently spatial. These include diffusion in complex spaces, sub-cellular localization, and transient membrane associations. Additionally, important cellular processes often rely on molecular species present in low numbers. Computer models that ignore these spatial and stochastic aspects of biological function clearly cannot offer insights into them. For example, non-spatial, non-stochastic models of the *E. coli* chemotaxis signaling network were invaluable for elucidating the basic system architecture [59] but could not aid investigation in the variation in signals received by different individual flagellar motors [28], the localization of molecules of the CheZ phosphatase [27],[60], or the formation of intracellular concentration gradients of the CheY signal transmitting protein [29]. Moreover, predictions about the behavior of systems where space and stochasticity are factors are approximate at best and may be qualitatively incorrect [61]. For example, a simple chemical oscillating system executes nearly sinusoidal oscillation when simulated without spatial or stochastic detail but exhibits intermittent boom and bust cycles when space and stochasticity are included [24]; also, spatial correlations can cause distributive multiple phosphorylation mechanisms, in which kinases release their substrates between phosphorylations, to lose ultrasensitivity to the substrate concentration [62].

We devised Smoldyn 2.1 to help address the need for accurate and efficient spatial stochastic simulation software. We designed it to simulate molecules and membranes, and events including diffusion, chemical reactions, adsorption, and desorption. We demonstrated the capabilities of Smoldyn with a model of signaling between yeast cells through a diffusible pheromone. The model suggested that, by degrading pheromone, the protease secreted by *MAT* **a** type yeast cells steepened the local pheromone concentration gradient, which helps cells locate and choose among potential mating partners.

On a contemporary laptop computer (2006 MacBook Pro) Smoldyn can perform useful simulations involving assemblages of more 100,000 molecules with relative ease. This power is sufficient to investigate many biochemical systems, including the *E. coli* chemotaxis signaling system and signaling between neurons. Extrapolating computer power with Moore's law, Smoldyn should be able to simulate all 2.6 million proteins in an *E. coli* cell [63] (or a mitochondrion or yeast nucleus, which are roughly the same size) within 5 years, still on a single laptop computer. Using more powerful computers, such as Beowulf clusters [64] or the current NVIDIA Tesla [65], Smoldyn should be able to simulate entire populations of complete cells over many generations, within a decade.

However, many challenges to simulations of entire cells and populations remain. First, neither Smoluchowski dynamics nor Green's Function Reaction Dynamics are wholly adequate. For that reason, researchers will need to develop new physical theories for reactions and diffusion in crowded cytoplasms, the mechanical interactions between cytoskeletal filaments and cell membranes, and the functions of extended macromolecular complexes. These theories, which may be partially empirical, need to isolate the essential behaviors of these processes so that they can be modeled. Second, these theories will need to be embodied in algorithms so that modelers can account for the corresponding processes in their cellular models. Third, not all aspects of cellular processes require attention to spatial and stochastic detail (for example, there are likely to be over a million ATP molecules in an *E. coli* cell [63], probably with weak spatial gradients), so multi-level algorithms, such as those that combine stochastic and deterministic methods [66], will be valuable. Fourth, although it is possible that there might be increasingly standardized and quantitative experiments that assist modeling of some cellular processes, almost by definition, the experiments on newly explored cellular processes will be diverse and incomplete. Any modeling effort that expects to contribute to the understanding of newly articulated cellular phenomena will require simulation software that can work with diverse and incomplete experimental results.

## Methods

We wrote the core portion of Smoldyn in the C programming language. This core is linked to the OpenGL library for graphics, the libtiff library for saving tiff format images, the libmoleculizer library for rule-based reaction network generation [32], the POSIX library for threaded operation, and the SIMD-oriented fast Mersenne Twister library [67] for random number generation. The combined program compiles and links on Macintosh OS X or Linux systems with the gcc compiler. Windows versions are cross-compiled from Macintosh using the mingw compiler. We used Valgrind to check for memory leaks and gprof for code profiling. All source code, makefiles, executable applications, example configuration files, utility programs, and documentation can be downloaded for free from www.smoldyn.org. The code is licensed under the Gnu General Public License.

## Supporting Information

Text S1 Additional information for Table 2
(0.53 MB PDF)

Text S2 Details for Figure 3
(0.61 MB PDF)

Text S3 Runtime scales linearly with the number of molecules
(0.12 MB PDF)

Text S4 Bar1 model details
(0.15 MB PDF)
