# Supplementary material for: Detailed Simulations of Cell Biology with Smoldyn 2.1
Source: PLoS Comput Biol. 2010 Mar 12;6(3):e1000705. doi: 10.1371/journal.pcbi.1000705 (PMC2837389; doi:10.1371/journal.pcbi.1000705)
Supplement: Text S1 — Additional information for Table 2 (0.53 MB PDF) [file pcbi.1000705.s001.pdf]

## Text S1: Additional information for Table 2

## Supplementary Information for
## Detailed simulations of cell biology with Smoldyn 2.1

Andrews, Addy, Brent, and Arkin

Table 2 compares the ChemCell, MCell, and Smoldyn spatial stochastic simulators. Most of the information presented here about ChemCell is derived from the "ChemCell Documentation" for the September 10, 2008 version of ChemCell. It was confirmed through discussions with Steve Plimpton, who is the ChemCell author. Most information about MCell is from Kerr et al., 2008 [1] or from the "MCell3 Quick Tutorial and Reference Guide," dated October 23, 2008. Most information about Smoldyn is also presented in the main text of this article or is repeated in the March 2009 version of the Smoldyn Documentation. Additional information is described and/or referenced below.

| feature | ChemCell | MCell3 | Smoldyn 2.0 |
|---|---|---|---|
| **simulation methods** | **ODE, Gillespie, particle** | **particle** | **particle** |

ChemCell can operate in any of three distinct modes: non-spatial deterministic (ODE simulations), non-spatial stochastic (Gillespie simulations), or particle-based simulations. ChemCell cannot use multiple modes simultaneously.

| | | | |
|---|---|---|---|
| **time steps** | **fixed** | **adaptive** | **fixed** |

Version 3 of MCell introduced a scheduling-based system in which "the scheduler maintains a user-specified canonical time step $\Delta T$ during the simulation, but allows an internal $\Delta t$ to vary from molecular species to molecular species" [1]. While not as sophisticated as Green's Function Reaction Dynamics [2], this scheduling-based system should nevertheless be much more computationally efficient than can be achieved with fixed time steps.

| | | | |
|---|---|---|---|
| **system dimensionality** | **3** | **3** | **1, 2, 3** |

Smoldyn can perform simulations in 1-, 2-, or 3-dimensional systems. Low-dimensional simulations can be useful, for example, for investigating protein diffusion along a nucleic acid filament or interaction rates of integral membrane proteins.

| | | | |
|---|---|---|---|
| **system boundaries** | **reflective, periodic** | **reflective, absorbing, transparent** | **reflective, absorbing, periodic, transparent** |

System boundaries are called periodic if molecules that diffuse out of one side of the system then diffuse into the opposite side of the system. Periodic boundaries are useful for efficiently simulating large homogeneous systems without incurring unwanted effects from reflective system boundaries. ChemCell and Smoldyn support periodic boundaries.

Transparent boundaries, which MCell and Smoldyn support, allow molecules to diffuse out of the system boundaries. Even outside the boundaries, these molecules are still tracked and they can still react or interact with surfaces.

| | | | |
|---|---|---|---|
| **spatial partitioning** | **constant size** | **variable size** | **constant size** |

The system volume needs to be spatially partitioned so that the programs can find bimolecular reaction partners efficiently, as well as to simulate molecule-surface interactions efficiently [3,4]. Each of these programs offers several options with which the user can choose the partition spacing. For example, depending on the program, the user can choose the partition sizes, the number of partitions, the partition locations, or the average molecules per partition. MCell and Smoldyn also include defaults so that the volume is spatially partitioned automatically, without any user input.

| | | | |
|---|---|---|---|
| **geometry primitives** | **triangles, spheres, boxes, planes, cylinders** | **triangles** | **triangles, rectangles, spheres, cylinders, hemispheres, disks** |

All three programs represent surfaces as lists of primitive surface objects. These are called "triangles" or "regions" in ChemCell, "elements" or "tiles" in MCell and "panels" in Smoldyn. Using triangle-shaped primitives, each program allows simulations with arbitrarily complex triangulated mesh surfaces, from which any shape can be constructed [5]. In addition, ChemCell supports spheres, boxes, planes, or axis-aligned cylinders as surface primitives. MCell does not support additional primitive shapes, although it allows the user to specify boxes in the configuration file for convenience, which it represents internally as triangles. Along with triangles, Smoldyn supports spheres, cylinders, disks, hemispheres, and axis-aligned rectangles. More surface primitives can simplify configuration file development and can speed up simulations.

| | | | |
|---|---|---|---|
| **surface molecule density** | **unlimited** | **up to 1 molecule /tile** | **unlimited** |

MCell only allows up to 1 molecule per surface element (triangular tile). When MCell simulates surface diffusion, these molecules have fixed point-like positions. However, when MCell simulates chemical reactions, it does not consider the exact positions of these molecules, but instead treats each molecule as though it is at a random position within its tile. These limitations, which are rarely important, can be minimized by constructing surfaces from very small tiles.

| | | | |
|---|---|---|---|
| **concentration fixing** | **no** | **near surfaces** | **on surfaces, in compartments** |

MCell permits the user to clamp the chemical concentration at a boundary. MCell absorbs all molecules of the appropriate type when they hit the boundary, and also creates other molecules of this type at the boundary at a constant rate. Smoldyn also allows fixed-concentration boundaries, although with a different method. Smoldyn permits the user to fix the number of surface-bound molecules that are on a surface to a constant number. Whenever this fixing is done, which may occur a single time point, periodically, or at every time step, Smoldyn adds or removes molecules, as required, to achieve the correct molecule count. Adsorption or desorption to or from the surface then allows the surface to act as a fixed-concentration sink or source. While ChemCell does not support fixed-concentration boundaries, its "fix" command is a flexible way for a programmer to add new boundary conditions, so these could be supported if they were needed.

| | | | |
|---|---|---|---|
| **surface molecule states** | **transmembrane** | **integral states: top-front, top-back** | **integral, peripheral states: up, down, front, back** |

In MCell, surface molecules may be oriented "top-front" or "top-back," to distinguish the two transmembrane orientations. Smoldyn supports these transmembrane orientations as well, which it calls "up" and "down." Smoldyn also permits molecules to be adsorbed to the front or back of a surface, called the "front" and "back" surface-bound states, respectively, which can represent peripheral membrane proteins. In ChemCell, all molecules are either in solution or are bound to a surface, with no further orientation detail. However, reactions can be made to depend on whether a surface-bound molecule is on the front or back of a surface. Thus, for example, transmembrane receptors can have different internal and external reactions, if all such receptors face the same direction; this does not work well if some receptors face inward and some face outward.

| | | | |
|---|---|---|---|
| **accuracy of diffusion** | **volume: exact**<br>**surface: approx.** | **volume: exact**<br>**surface: approx.** | **volume: exact**<br>**surface: approx.** |

ChemCell can diffuse molecules with any of several algorithms, of which the most accurate performs Gaussian-distributed random displacements of molecules on each spatial coordinate at each time step. Smoldyn implements this algorithm as well. MCell diffuses molecules by choosing a random direction and a random distance from the appropriate probability distribution. These two methods are equivalent and each is exact for any length simulation time step.

MCell diffuses each surface-bound molecule by choosing a random displacement vector that is within the plane of the local surface tile, and then bending this vector as needed as it crosses onto adjacent tiles. This method is exact within single tiles and for nearest-neighbor tiles, but is in error for longer diffusive steps [1]. Smoldyn diffuses each surface-bound molecule by diffusing it in three dimensions and then moving it back to the surface along the local surface normal, which is slightly less accurate than MCell's method. ChemCell uses a combination of methods: it uses the MCell method for triangular surface tiles and it diffuses in the 2-D plane of the surface and then projects back to the surface for curved surface regions, which is similar to the Smoldyn method (Plimpton, personal communication, 2009). For all methods, surface diffusion is nearly exact if the average diffusive step lengths of molecules is substantially less than the surface's radius of curvature.

| | | | |
|---|---|---|---|
| **accuracy of reactions in solution** | **order 0: none**<br>**order 1: exact**<br>**order 2: approx.** | **order 0: none**<br>**order 1: exact**<br>**order 2: approx.** | **order 0: exact**<br>**order 1: exact**<br>**order 2: exact** |

All programs simulate unimolecular and bimolecular reactions. In addition, Smoldyn supports order 0 reactions, in which molecules are generated at a constant rate, and are placed at random positions within the system volume, or within a sub-compartment. These can be useful for representing, for example, protein synthesis but not the entire synthetic machinery.

Bimolecular reactions are handled differently by each program. In ChemCell, if two molecules end up within a user-chosen cut-off distance at the end of a time step, and a random number is less than a pre-determined reaction probability, then

they react. ChemCell calculates this reaction probability under the assumption that reactants are well-mixed, rather than at steady-state. As described below, this can cause bimolecular reactions to simulate with too slow a reaction rate. ChemCell can also run reactions with unit reaction probability and a user-chosen cut-off distance; if the user finds this cut-off distance with the Smoldyn program, it can be used in ChemCell to yield accurate reaction rates.

In MCell, molecules are assumed to travel in straight lines during time steps. As a molecule travels, it is able to interact with any molecule whose center is within a molecular diameter of this trajectory. For each of these potential interactions, MCell decides whether a bimolecular reaction should occur based on whether a random number is less than the appropriate reaction probability. As with the ChemCell algorithm, these reaction probabilities were calculated with the assumption of well-mixed reactants. We show below that this can cause MCell to simulate bimolecular reactions with reaction rates that are too slow.

In Smoldyn, a bimolecular reaction occurs if two reactants end up within a pre-determined "binding radius" of each other at the end of a time step, and not otherwise. This is similar to the algorithm used by ChemCell, but differs in the fact that reactions always occur upon molecule interactions, rather than with some fixed probability. The Smoldyn binding radius is calculated for a steady-state reactant distribution, rather than a well-mixed one, which yields more accurate reaction rates.

We investigated the bimolecular reaction rate accuracy of the three simulators by simulating the generic reaction A + B → C. If the system starts with the same number of A and B molecules, then the non-spatial mass action reaction rate equations lead to the solution

$$n_A(t) = \left[ \frac{1}{n_A(0)} + \frac{kt}{V} \right]^{-1}$$

Here, $n_A(t)$ is the number of A molecules as a function of time, $k$ is the reaction rate constant, $t$ is the time, and $V$ is the system volume. Our simulations started with 8000 molecules of each A and B, which started at random locations within the system volume (for the Smoldyn simulations, the molecule starting positions were again completely random, except that no A-B pairs were allowed to start closer than a binding radius of each other). Systems were cubical with 0.2 μm long sides. Each simulation actually ran three simulations in parallel, with their own A and B molecules: one with a slow reaction rate, $5.88\times10^5$ $M^{-1}s^{-1}$, one with a medium reaction rate, $5.88\times10^6$ $M^{-1}s^{-1}$, and one with a fast reaction rate, $5.88\times10^7$ $M^{-1}s^{-1}$ (these rates convert to 1, 10, and 100 $nm^3$/μs, respectively). The complete configuration files for these simulations are shown below.

In addition, we ran these simulations with two different time steps, a step length of 0.1 μs and another of 0.01 μs. As shown in the figures below, all programs performed in good agreement with the mass action theory for the slow reaction

rate. This was true for either the longer or shorter time steps (although ChemCell is slightly too slow with short time steps). For the medium reaction rates, ChemCell and MCell reactions were significantly slower than the mass action theory, while Smoldyn agreed reasonably well. For the fast rate, ChemCell and MCell were much slower yet, while Smoldyn showed fair agreement.

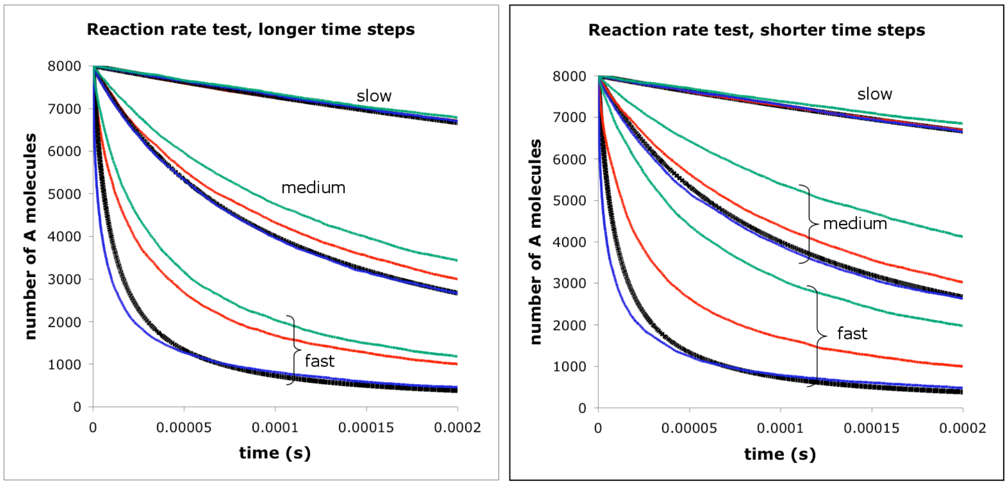


**Figure caption.** Number of A molecules in the system as a function of time for $A + B \rightarrow C$ reaction. Each panel shows lines for mass action theory (bold black lines), ChemCell (green), MCell (red), and Smoldyn (blue). Each panel also shows lines for three different reaction rates, which are named slow, medium, and fast. For the right panel, simulation time steps were 1/10th the size of those that were used for the left panel.

For the fast rate (and, to a lesser extent, the medium rate with short time steps), Smoldyn appears to be too fast initially. This is actually not a fault of Smoldyn, but reflects the fact that mass action kinetics do not accurately describe the short-time behavior of diffusion-influenced reactions [6]. In particular, the reaction rate "constant" is not actually constant when reactions are diffusion-influenced, but is time-dependent during short times. In this time regime, Smoldyn is more accurate than is the mass action theory. At longer times, still with the fast reaction rates, the Smoldyn reactions are slightly too slow. Again, the difference arises from subtle aspects of diffusion-influenced reaction rates. This time, it arises from the fact that the reaction rate "constant" also depends on the relative concentrations of the two chemical species. The Smoluchoski theory, on which we derived the Smoldyn reaction parameters, only strictly applies to the situation in which there are many more A molecules than B molecules, or vice versa [6]. However, there are the same number of each reactant in this simulation, which leads to small errors. The following figure shows a Smoldyn simulation that is identical to the fast one, shown with the shorter time steps, except that the system was started with only 4000 B molecules. Note that agreement is much better for this situation.

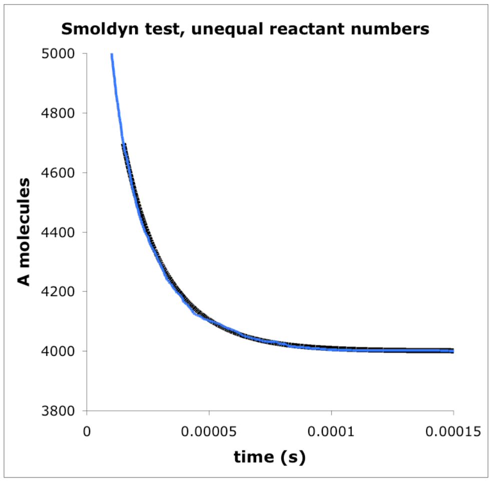


**Figure caption.** This figure is identical in all ways to the Smoldyn test that is shown above, with short time steps and for the fast reaction rate, except that we reduced the starting number of B molecules from 8000 to 4000. Again, the bold black line shows the mass action theory solution (now starting at time 15 µs to avoid the transient early dynamics) and the blue line shows the simulation solution. Here, the simulated reaction rate is in essentially perfect agreement with the theoretical reaction rate because of the unequal numbers of A and B molecules.

## ChemCell configuration file

```
# Michaelis-Menten reaction
# ChemCell file

run_style     spatial
timestep      1e-7

global        0 0 0 0.2 0.2 0.2
boundary      p p p
move_style    cube brownian

species       A
species       B
species       C

reaction      slow A B 5.88e5 C
#reaction     med A B 5.88e6 C
#reaction     fast A B 5.88e7 C

diffusion     * 1e-8

bin           size 0.01 0.01 0.01

include       bireactccellpart.txt
stats         1

run           2000
```

bireactccellpart.txt

```
# ChemCell file

particles A 8000
1    0.052762162   0.177788884   0.187955221
2    0.062832008   0.00891862    0.064012626
3    0.162923843   0.031756133   0.097959302
4    0.140428646   0.046248355   0.013947209
...
7998 0.167887989   0.130943919   0.051917036
7999 0.050976038   0.153754829   0.038985047
8000 0.024745831   0.069111126   0.143708234

particles B 8000
1    0.133160688   0.070426388   0.198324634
2    0.113778752   0.031379865   0.13329166
3    0.114084279   0.132937218   0.057251039
...
7999 0.141369559   0.004928443   0.14891238
8000 0.037297363   0.138519329   0.153804316
```

## Smoldyn configuration file

```
# Bimolecular reactions
# units are nm, us

graphics none

dim 3
max_names 10
name As
name Bs
name Cs
name Am
name Bm
name Cm
name Af
name Bf
name Cf

max_mol 70000

molecule_lists slow med fast prod
mol_list As slow
mol_list Bs slow
mol_list Cs prod
mol_list Am med
mol_list Bm med
mol_list Cm prod
mol_list Af fast
mol_list Bf fast
mol_list Cf prod
```

```
difc all 1

time_start -0.1
time_stop 200
time_step 0.1

boundaries 0 0 200 p
boundaries 1 0 200 p
boundaries 2 0 200 p

mol 8010 As u u u
mol 8010 Bs u u u
mol 8100 Am u u u
mol 8100 Bm u u u
mol 12500 Af u u u
mol 12500 Bf u u u

output_files bireactABout.txt
cmd j 1 1 1 fixmolcount As 8000
cmd j 1 1 1 fixmolcount Bs 8000
cmd j 1 1 1 fixmolcount Am 8000
cmd j 1 1 1 fixmolcount Bm 8000
cmd j 1 1 1 fixmolcount Af 8000
cmd j 1 1 1 fixmolcount Bf 8000
cmd e molcount bireactABout.txt

reaction slow As + Bs -> Cs 1
reaction med  Am + Bm -> Cm 10
reaction fast Af + Bf -> Cf 100

end_file
```

## MCell configuration file

```
/* irreversible bimolecular reactions */

dt = 1.0e-7
iterations = 2000

TIME_STEP = dt
ITERATIONS = iterations
INTERACTION_RADIUS = 0.003

NOTIFICATIONS {
   ALL_NOTIFICATIONS = ON
}

WARNINGS {
   ALL_WARNINGS = ERROR
}


DEFINE_MOLECULES
{
   As {DIFFUSION_CONSTANT_3D = 1e-8}
   Bs {DIFFUSION_CONSTANT_3D = 1e-8}
   Cs {DIFFUSION_CONSTANT_3D = 1e-8}
   Am {DIFFUSION_CONSTANT_3D = 1e-8}
   Bm {DIFFUSION_CONSTANT_3D = 1e-8}
   Cm {DIFFUSION_CONSTANT_3D = 1e-8}
   Af {DIFFUSION_CONSTANT_3D = 1e-8}
   Bf {DIFFUSION_CONSTANT_3D = 1e-8}
   Cf {DIFFUSION_CONSTANT_3D = 1e-8}
}

DEFINE_REACTIONS
{
   As + Bs -> Cs [5.88e5]:rxn_s
   Am + Bm -> Cm [5.88e6]:rxn_m
   Af + Bf -> Cf [5.88e7]:rxn_f
}

INSTANTIATE world OBJECT
{
   system BOX {
      CORNERS = [0,0,0],[0.2,0.2,0.2]
      DEFINE_SURFACE_REGIONS {
         all {
            ELEMENT_LIST = [ALL_ELEMENTS]
         }
      }
   }

   As_release_site RELEASE_SITE {
      SHAPE = world.system[all]
      MOLECULE = As
      NUMBER_TO_RELEASE = 8000
   }

   Bs_release_site RELEASE_SITE {
      SHAPE = world.system[all]
      MOLECULE = Bs
      NUMBER_TO_RELEASE = 8000
   }

   Am_release_site RELEASE_SITE {
      SHAPE = world.system[all]
      MOLECULE = Am
      NUMBER_TO_RELEASE = 8000
   }

   Bm_release_site RELEASE_SITE {
      SHAPE = world.system[all]
      MOLECULE = Bm
      NUMBER_TO_RELEASE = 8000
   }

   Af_release_site RELEASE_SITE {
      SHAPE = world.system[all]
      MOLECULE = Af
      NUMBER_TO_RELEASE = 8000
   }

   Bf_release_site RELEASE_SITE {
      SHAPE = world.system[all]
      MOLECULE = Bf
      NUMBER_TO_RELEASE = 8000
   }
}

REACTION_DATA_OUTPUT {
   STEP = dt * 1
   {COUNT[As,WORLD]}=>"./reaction_data/As.dat"
   {COUNT[Bs,WORLD]}=>"./reaction_data/Bs.dat"
   {COUNT[Cs,WORLD]}=>"./reaction_data/Cs.dat"
   {COUNT[Am,WORLD]}=>"./reaction_data/Am.dat"
   {COUNT[Bm,WORLD]}=>"./reaction_data/Bm.dat"
   {COUNT[Cm,WORLD]}=>"./reaction_data/Cm.dat"
   {COUNT[Af,WORLD]}=>"./reaction_data/Af.dat"
   {COUNT[Bf,WORLD]}=>"./reaction_data/Bf.dat"
   {COUNT[Cf,WORLD]}=>"./reaction_data/Cf.dat"
}
```

| **accuracy of reactions** | **order 0: none** | **order 0: none** | **order 0: exact** |
|---|---|---|---|

**on surfaces order 1: exact order 1: exact order 1: exact**
**order 2: not quantitative order 2: approx. order 2: not quantitative**

All three simulators are able to simulate reactions between pairs of surface-bound molecules. ChemCell and Smoldyn use the same algorithms for surface reactions as for volume reactions. These are quantitatively inaccurate because they ignore the dimensionality difference. This error is most clearly seen by the fact that surface reaction rate constants should have units of area/time, whereas those for volume reactions should have units of volume/time (*e.g.* $M^{-1}s^{-1}$). In contrast, MCell implements a reasonably accurate surface reaction algorithm [1]. Nevertheless, it is approximate because it does not fully account for reactant diffusion [1]; it assumes a well-mixed reactant distribution, rather than a steady-state one.

**dissociation reaction at reactants, not stochastic, for microscopic fixed separation, for**
**product placement quantitative reversibility accurate reaction rates**

A geminate recombination occurs when a molecular complex dissociates to two molecules, and then those two molecules recombine to form a complex again. Smoldyn accounts for the effect of geminate recombinations on overall reaction rates by initially separating dissociation products by a fixed "unbinding radius." This produces accurate reaction rates and equilibrium concentrations. MCell accounts for geminate recombinations by allowing simulations to run with a "microscopic reversibility" feature [1]. When this is activated, dissociation products are initially separated by a random distance that is chosen from a probability density which is calculated to match that of the reactants before they combined to form a complex.

**location-specific reactions no surfaces surfaces, compartments**

MCell and Smoldyn allow the user to specify that certain reactions can only take place at a specific surface. ChemCell does not allow this, but makes it relatively easy to create different molecular species on different surfaces, which can then undergo different reactions.

In Smoldyn, the user can also state that certain reactions only take place within specific compartments. This way, for example, a protein may decompose when it is within the cell but not outside of it, or vice versa. ChemCell does not explicitly support compartment-specific reactions, but it does allow molecular species to change as they diffuse through surfaces (as does Smoldyn), which could be used for different reactions in different compartments.

**conformational spread no no yes**
**reactions**

Smoldyn supports conformational spread reactions, which are a special type of bimolecular reactions. Here, each reactant is replaced by a product, and these products are placed at the reactant locations. This may be useful for simulating the spread of activity across a tightly coupled cluster of receptor proteins [7]. ChemCell supports several options for the placement of products following a reaction, but not this one.

| | | | |
|---|---|---|---|
| **surface interactions** | **adsorb: not quantitative**<br>**desorb: exact**<br>**perm.: not quantitative** | **adsorb: not quantitative**<br>**desorb: exact**<br>**perm.: not quantitative** | **adsorb: exact**<br>**desorb: exact**<br>**permeable: exact** |

All three programs use the same basic algorithm to adsorb molecules to surfaces. If a molecule diffused across a surface during the previous time step, it adsorbs to the surface according to a pre-computed probability. The programs differ on how they calculate the probability value. ChemCell does not try to compute it, but requires the user to enter it. MCell calculates the probability by treating adsorption like a reaction between a volume molecule and a surface-bound molecule. As a result, the "rate constant" for adsorption in MCell has units of $M^{-1}s^{-1}$, which does not match the length/time units that ought to be used for adsorption coefficients. However, if MCell's adsorption reaction rate constant is divided by the area for each surface tile, then the MCell probability equation (ref. [1] eq. 4.7) matches the analogous probability that was derived by Erban and Chapman (ref. [8] eq. 10). This derivation assumes a well-mixed molecular distribution. Smoldyn achieves essentially exact adsorption rates by calculating the adsorption probability for a steady-state molecular distribution [9]. The Smoldyn algorithm also explicitly accounts for the differences between irreversible and reversible adsorption.

All three programs calculate the probability that a molecule desorbs from a surface during a time step with the same algorithm that they use for unimolecular reactions. This simulates the molecular concentrations on the surface and in solution exactly. To also yield accurate molecular spatial distributions, Smoldyn desorbs molecules with an initial separation between the desorbed molecule and the surface; this separation is randomly chosen from probability densities for reversible or irreversible desorption, as appropriate.

| | | | |
|---|---|---|---|
| **compartments** | **no** | **somewhat** | **yes** |

Smoldyn allows the user to define spatial compartments within the simulation volume. For example, these might be a nucleus, cytoplasm, or extra-cellular space, or just the left and right halves of a cell. Compartments are useful for compartment-specific reactions, described above, for initial molecule placement, for data output, and for linking Smoldyn to other simulators. MCell does not support compartments to the same extent, but does allow molecule counts to be reported inside specified spatial objects or regions.

| | | | |
|---|---|---|---|
| **parallel processing** | **MPI** | **MPI** | **POSIX threads** |

ChemCell [10], MCell [11], and Smoldyn are able to take advantage of multi-processor computers. ChemCell and MCell are distributed-memory programs that use MPI function calls. These allow them to run on most parallel computers. In addition, ChemCell supports embarrassingly parallel operation such that one input script can be easily run on multiple processors in parallel for statistical sampling purposes (Plimpton, personal communication, 2009). Smoldyn cannot take advantage of distributed-memory systems currently, but can instead run in multiple threads at once. This speeds operation on multi-processor and multi-core

computers. We have not optimized this feature yet, so it does not actually provide substantial speed improvements at present.

| **graphics** | **post-run with pizza.py** | **post-run with DReAMM** | **during simulation** |
|---|---|---|---|

All three programs allow graphical output of results. ChemCell and MCell save visualization data during the simulation, which can be viewed later but not during the simulation. Smoldyn presents a graphical display during the simulation, but does not save the visualization data for future analysis.

ChemCell graphics can be viewed in the python program called pizza.py, while MCell visualization data can be viewed in the OpenDX DReAMM program (Design, Render, and Animate MCell Models). We found both visualization programs to be challenging to install. Smoldyn does not use a separate visualization program to render graphics.

| **configuration file language** | **math geometry manipulations** | **math geometry manipulations** | **none** |
|---|---|---|---|

Both ChemCell and MCell allow the user to specify simple mathematical operations within the configuration file. In these programs, the user may define variables and then manipulate them with algebra or simple functions such as sin($x$) and log($x$). This feature can simplify the development and reuse of configuration files. While much less powerful, Smoldyn offers a "define" statement, which performs simple text-replacement.

MCell also allows the user to specify translation and rotation transformations for geometrical objects. Again, this can simplify the development and reuse of configuration files.

| **source code** | **open, GPL license** | **closed** | **open, GPL license** |
|---|---|---|---|

All three programs are free and are available on the web. ChemCell is at http://www.sandia.gov/~sjplimp/chemcell.html, MCell is at: http://www.mcell.cnl.salk.edu, and Smoldyn is at http://www.smoldyn.org. MCell requires user registration and has closed source code. ChemCell and Smoldyn do not require registration and are licensed under the Gnu General Public License.

| **benchmark run time** | **99 s** | **120 s** | **47 s** |
|---|---|---|---|

We ran identical simulations on all three simulators to compare their run times. These simulations perform the simple Michaelis-Menten enzymatic reaction

$$E + S \underset{back}{\overset{fwd}{\rightleftharpoons}} ES \xrightarrow{prod} E + P$$

The reaction rates were $k_{fwd}$ = 5.88e6 $M^{-1}s^{-1}$ (0.01 $\mu m^3/s$), $k_{back} = 1\ s^{-1}$, and $k_{prod} = 1\ s^{-1}$; all species were assigned a diffusion coefficient of 10 $\mu m^2 s^{-1}$; simulations ran for 10 s of simulated time with a time step of 0.001 s, so each simulator performed 10,000 iterations; systems were cubical with each side equal to 4.4964 $\mu m$ for a total volume of 90.9 $\mu m^3$; and simulations were started with 909

randomly placed E molecules (10 μm$^{-3}$) and 9091 randomly placed S molecules (100 μm$^{-3}$), which add to a total of 10,000 starting molecules. As much as possible, we optimized the simulator settings to yield the fastest times possible. The setting that made the most difference was the size of the spatial partitions. Smoldyn was fastest with 15 partitions in each dimension, MCell was fastest with 11 partitions in each dimension, and ChemCell was fastest with 16 bins in each dimension.

All simulators yielded nearly identical results, shown in the figure. Black lines represent mass action theory, green are ChemCell results, red are MCell results, and blue are Smoldyn results.

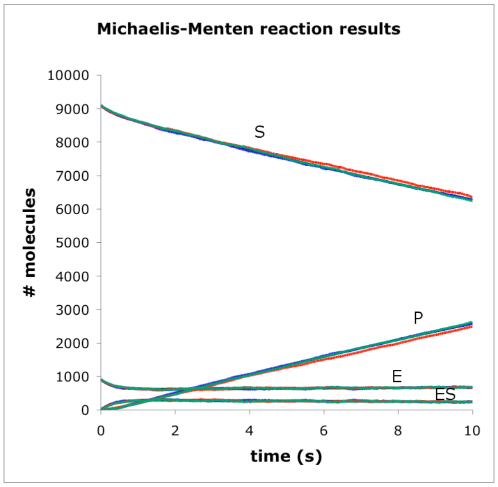


The configuration files for these tests are:

<u>ChemCell</u>

```
# Michaelis-Menten reaction
# ChemCell file

run_style     spatial
timestep      0.001

global        0 0 0 4.4964 4.4964 4.4964
boundary      p p p
move_style    cube brownian

species       E
species       S
species       ES
species       P

reaction      fwd E S 5.88e6 ES
reaction      back ES 1.0 E S
reaction      prod ES 1.0 E P

diffusion     * 1e-7

bin           diff 1e-7 rcount 4 4 4

include       benchmarkccellpart.txt

stats         10

run           10.0
```

<u>MCell</u>

```
/* Michaelis-Menten simulation */

dt = 1.0e-3
iterations = 10000
boxsize = 4.4964
partitions = 11

TIME_STEP = dt
ITERATIONS = iterations
INTERACTION_RADIUS = 0.003

NOTIFICATIONS {
   ALL_NOTIFICATIONS = ON
}

WARNINGS {
   ALL_WARNINGS = ERROR
}

PARTITION_X = [[0 TO boxsize STEP boxsize/partitions]]
```

```
PARTITION_Y = [[0 TO boxsize STEP boxsize/partitions]]
PARTITION_Z = [[0 TO boxsize STEP boxsize/partitions]]

DEFINE_MOLECULES
{
   E {DIFFUSION_CONSTANT_3D = 1e-7}
   S {DIFFUSION_CONSTANT_3D = 1e-7}
   ES {DIFFUSION_CONSTANT_3D = 1e-7}
   P {DIFFUSION_CONSTANT_3D = 1e-7}
}

DEFINE_REACTIONS
{
   E + S -> ES [5.88e6]:rxn_fwd
   ES -> E + S [1]:rxn_back
   ES -> E + P [1]:rxn_prod
}

INSTANTIATE world OBJECT
{
   system BOX {
      CORNERS = [0,0,0],[boxsize,boxsize,boxsize]
      DEFINE_SURFACE_REGIONS {
         all {
            ELEMENT_LIST = [ALL_ELEMENTS]
         }
      }
   }

   E_release_site RELEASE_SITE {
      SHAPE = world.system[all]
      MOLECULE = E
      NUMBER_TO_RELEASE = 909
   }

   S_release_site RELEASE_SITE {
      SHAPE = world.system[all]
      MOLECULE = S
      NUMBER_TO_RELEASE = 9091
   }
}

REACTION_DATA_OUTPUT {
   STEP = dt * 10
   {COUNT[E,WORLD]}=>"./reaction_data/E.dat"
   {COUNT[S,WORLD]}=>"./reaction_data/S.dat"
   {COUNT[ES,WORLD]}=>"./reaction_data/ES.dat"
   {COUNT[P,WORLD]}=>"./reaction_data/P.dat"
}
```

## Smoldyn

```
# Michaelis-Menten reaction
# units: micron and second

graphics none

dim 3
names E S ES P
max_mol 11000
molperbox 3
accuracy 10

difc E 10
difc S 10
difc ES 10
difc P 10

time_start 0
time_stop 10
time_step 0.001

boundaries 0 0 4.4964 p
boundaries 1 0 4.4964 p
boundaries 2 0 4.4964 p

molecule_lists Elist Slist ESlist Plist
mol_list E Elist
mol_list S Slist
mol_list ES ESlist
mol_list P Plist

output_files benchmarkout.txt
cmd i 0 100 0.01 molcount benchmarkout.txt

# 10 E/vol and 100 S/vol
mol 909 E u u u
mol 9091 S u u u

reaction fwd E + S -> ES 0.01
reaction back ES -> E + S 1
reaction prod ES -> E + P 1
product_placement back pgemmax 0.2

end_file
```

## References


1. Kerr RA, Bartol TM, Kaminsky B, Dittrich M, Chang J-CJ, et al. (2008) Fast Monte Carlo simulation methods for biological reaction-diffusion systems in solution and on surfaces. SIAM J Sci Comput 30: 3126-3149.
2. van Zon JS, ten Wolde PR (2005) Green's function reaction dynamics: A particle-based approach for simulating biochemical networks in time and space. J Chem Phys 123: 234910.
3. Stiles JR, Bartol TM (2001) Monte Carlo methods for simulating realistic synaptic microphysiology using MCell. In: De Schutter E, editor. Computational Neuroscience: Realistic Modeling for Experimentalists. Boca Raton, FL: CRC Press. pp. 87-130.
4. Andrews SS, Bray D (2004) Stochastic simulation of chemical reactions with spatial resolution and single molecule detail. Phys Biol 1: 137-151.
5. Coggan JS, Bartol TM, Esquenazi E, Stiles JR, Lamont S, et al. (2005) Evidence for ectopic neurotransmission at a neuronal synapse. Science 309: 446-451.
6. Keizer J (1987) Diffusion effects on rapid bimolecular chemical reactions. Chem Rev 87: 167-180.
7. Duke TAJ, LeNovère N, Bray D (2001) Conformational spread in a ring of proteins: a stochastic approach to allostery. J Mol Biol 308: 541-553.
8. Erban R, Chapman SJ (2007) Reactive boundary conditions for stochastic simulations of reaction-diffusion processes. Phys Biol 4: 16-28.
9. Andrews SS (2009) Accurate particle-based simulation of adsorption, desorption, and partial transmission. Phys Biol 6: 46015.
10. Plimpton SJ, Slepoy A (2005) Microbial cell modeling via reacting diffusive particles. J Phys: Conf Ser 16: 305-309.
11. Casanova H, Bartol TMJ, Stiles J, Berman F (2001) Distributing MCell simulations on the grid. Int J High Performance Comput 15: 243-257.
