# Supplementary material for: Detailed Simulations of Cell Biology with Smoldyn 2.1
Source: PLoS Comput Biol. 2010 Mar 12;6(3):e1000705. doi: 10.1371/journal.pcbi.1000705 (PMC2837389; doi:10.1371/journal.pcbi.1000705)
Supplement: Text S2 — Details for Figure 3 (0.61 MB PDF) [file pcbi.1000705.s002.pdf]

<u>Text S2: Details for Figure 3</u>
Supplementary Information for
Detailed simulations of cell biology with Smoldyn 2.1
Andrews, Addy, Brent, and Arkin

2.1 Figure 3, panel A.

The main panel shows $\langle r^2\rangle$ versus time for various diffusion coefficients, where r^2 is the squared distance between a molecule's position and the origin. It is well known [1] that the expectation value of r^2 for a random walk is $2n_DDt$, where n_D is the system dimensionality, D is the isotropic diffusion coefficient, and t is the time. Using this, the theoretical lines that are shown are

$$\langle r^2\rangle = 6Dt$$

This equation is also derived below.

The inset panel shows the ratio $\langle r^2\rangle/(6Dt)$. Here, we calculate the expectation and variance of this ratio, shown with solid and dashed lines in the figure, respectively. Consider 1 particle in 1 dimension, starting at the origin. The probability density of its location after time t is the Gaussian [2],

$$p(x=a) = \frac{1}{\sigma\sqrt{2\pi}}\exp\left(-\frac{a^2}{2\sigma^2}\right)$$

where $\sigma = (2Dt)^{1/2}$, D is the diffusion coefficient, and a is just a dummy variable. The probability density of x^2 is

$$p(x^2=a) = \frac{1}{2\sqrt{a}}p\left(x=\pm\sqrt{a}\right) = \frac{1}{\sqrt{a}}p\left(x=\sqrt{a}\right) = \frac{1}{\sqrt{a}}\cdot\frac{1}{\sigma\sqrt{2\pi}}\exp\left(-\frac{a}{2\sigma^2}\right)$$

The first equality is valid for any probability density, the second is valid for any even probability density, and the third is just from substitution. Integration from 0 to infinity verifies that the probability density is normalized. The expectation value and variance of x^2 are respectively

$$\langle x^2\rangle = \int_0^\infty p(x^2=a)\,a\,da = \sigma^2 = 2Dt$$

$$V(x^2) = \langle x^4\rangle - \langle x^2\rangle^2$$

The former term in the variance equation is

$$\langle x^4 \rangle = \int_0^\infty p(x^2 = a) a^2 da = 3\sigma^4 = 12D^2t^2$$

Thus, for a one-dimensional system, the expectation value, variance and standard deviation of x^2 are, respectively,

$$E(x^2) = 2Dt \qquad V(x^2) = 8D^2t^2 \qquad S.D.(x^2) = 2\sqrt{2}Dt$$

Now consider three dimensions, where motion on each of the three coordinates is independent of motion on the other coordinates. Again, one particle starts at the origin and then diffuses to location (x,y,z), which has distance r from the origin. Some identities are

$$\langle r^2 \rangle = \langle x^2 + y^2 + z^2 \rangle = \langle x^2 \rangle + \langle y^2 \rangle + \langle z^2 \rangle = 3\langle x^2 \rangle = 6Dt$$
$$\langle r^4 \rangle = \left\langle \left(x^2 + y^2 + z^2\right)^2 \right\rangle = \langle x^4 + y^4 + z^4 + 2x^2y^2 + 2x^2z^2 + 2y^2z^2 \rangle$$
$$= 3\langle x^4 \rangle + 6\langle x^2 \rangle^2 = 60D^2t^2$$

The former result is the one that was shown initially without proof. From the latter result, the variance and standard deviation of $\langle r^2 \rangle$ are found,

$$V(r^2) = \langle r^4 \rangle - \langle r^2 \rangle^2 = 24D^2t^2$$
$$S.D.(r^2) = 2\sqrt{6}Dt$$

To find the relative fluctuations, r^2 is divided by the expectation value to yield

$$E\left(\frac{r^2}{6Dt}\right) = 1 \qquad V\left(\frac{r^2}{6Dt}\right) = \frac{2}{3} \qquad S.D.\left(\frac{r^2}{6Dt}\right) = \sqrt{\frac{2}{3}}$$

Finally, the standard deviation is divided by $n^{1/2}$, where n is the number of molecules, to yield the theory values that are shown in the panel A inset.

$$S.D.\left(\frac{\langle r^2 \rangle}{6Dt}\right) = \sqrt{\frac{2}{3n}}$$

Following is the Smoldyn configuration file that was used to create the simulation data shown in panel A:

```
# Test of isotropic diffusion rate

graphics opengl
graphic_iter 10
```

```
dim 3
names red green blue
max_mol 3000
boxsize 200

difc red 100
difc green 10
difc blue 1

color red 1 0 0
color green 0 1 0
color blue 0 0 1

time_start 0
time_stop 100
time_step 0.1

boundaries 0 -100 100 t
boundaries 1 -100 100 t
boundaries 2 -100 100 t

mol 1000 red 0 0 0
mol 1000 green 0 0 0
mol 1000 blue 0 0 0

output_files diffioutr.txt diffioutg.txt diffioutb.txt
cmd e molmoments red diffioutr.txt
cmd e molmoments green diffioutg.txt
cmd e molmoments blue diffioutb.txt

end_file
```

2.2 Figure 3, panel B

The main panel shows the number of molecules surviving as a function of time, using three different first-order decay rates. These reactions follow the general reaction equation A → Ø. The mass action kinetics for the loss of reactant are described with the differential equation

$$\frac{d[\mathrm{A}]}{dt} = -k[\mathrm{A}]$$

where k is the first order reaction rate constant. This is solved to yield the deterministic solution for the number of A molecules as a function of time,

$$n(t) = n_0 e^{-kt}$$

n_0 is the number of A molecules at time 0 and $n(t)$ is the number at time t. This equation is used for the theory lines in the main panel.

Consider a reasonably narrow time slice during one of these decay reactions that is of size Δt. Here, narrow means that $\Delta t \ll \tau$, where $\tau = 1/k$, which is the characteristic

time of the reaction. The number of decays that is expected that occur during this time slice is

$$n_{rxn} = -\frac{dn(t)}{dt} \cdot \Delta t = n(t) k \Delta t$$

Reduced time is defined as $t' = t/\tau$ and a reduced time slice is $\Delta t' = \Delta t/\tau$. The expected number of decays in a reduced time slice is

$$n'_{rxn} = n(t) k \Delta t' \tau = n(t) \Delta t'$$

These are independent events, so they are Poisson distributed. Since n_{rxn}' is the expected number of events and the variance of a Poissonian is the same as the mean, n_{rxn}' is also the variance and $n_{rxn}'^{1/2}$ is the standard deviation for the number of events.

Graphed in the panel inset is the reduced reaction rate (RRR), for which the expectation value and standard deviation are respectively

$$E(RRR) = \frac{n'_{rxn}}{n(t)\Delta t'} = 1$$

$$S.D.(RRR) = \frac{\sqrt{n'_{rxn}}}{n(t)\Delta t'} = \frac{1}{\sqrt{n(t)\Delta t'}} = \frac{1}{\sqrt{n_0 \Delta t'}} \exp\left(\frac{t'}{2}\right)$$

The reduced reaction rate is the number of reactions per surviving molecule, per unit of reduced time.

Following is the configuration file used to produce the simulation data in panel B:

```
# Unimolecular reactions, rate testing

graphics opengl

dim 3
names red green blue
max_mol 15000

difc all 1

color red 1 0 0
color green 0 1 0
color blue 0 0 1

time_start 0
time_stop 50
time_step 0.01

boundaries 0 0 100 r
boundaries 1 0 100 r
boundaries 2 0 100 r
```

```
mol 1000 red u u u
mol 1000 green u u u
mol 1000 blue u u u

output_files unireact1out.txt
cmd e molcount unireact1out.txt

reaction slow red -> 0 0.1
reaction med green -> 0 1
reaction fast blue -> 0 10

end_file
```

Simulation data points in the panel inset are independent of each other, so we used Pearson's chi-squared test to evaluate the null hypothesis that the theory and the data represent the same distribution. This uses the equation

$$X^2 = \sum_{i=1}^{n} \left(\frac{y_i' - E_{RRR}(t_i)}{S.D._{RRR}(t_i)} \right)^2$$

where there are n data points, t_i are the time points, y'_i are the simulated reduced reaction rates, and other variables are defined as above. Comparison with a cumulative χ^2 distribution showed that 76%, 46%, and 96% of the χ^2 distribution exceeded the X^2 values that we found for the k = 0.1 s^{-1}, 1 s^{-1}, and 10 s^{-1} data sets, respectively. Because these percentages would have to be less than 5% for us to reject the null hypothesis to a 95% certainty level, this indicates that there are no statistically significant differences between the simulation data and the analytical theory.

2.3 Figure 3, panel C

The simulation data shown here are for the bimolecular reaction A + B → Ø, with the same reaction and same parameters as is presented in Figure 7 of Andrews and Bray [3]. Other than the trivial renaming of AH to A, the sole differences between this panel and that figure are the random numbers used and the fact that the y-axis here shows the number of molecules instead of the fraction of molecules. From equation 43 of Andrews and Bray, the expectation number of A molecules as a function of time is

$$n_A(t) = n_{A,0} \exp\left[-4\pi\sigma_b D \frac{n_B}{V} \left(1 + \frac{2\sigma_b}{\sqrt{\pi D t}} \right) t \right]$$

Parameters: $n_A(t)$ is the number of A molecules over time, $n_{A,0}$ is the initial number of A molecules, σ_b is the binding radius, D is the mutual diffusion coefficient, n_B is the number of B molecules (the simulation uses 60 times more B molecules than A molecules, so it is assumed in the theory that the number of B molecules is constant throughout the simulation), and V is the system volume. This equation is graphed in the main panel.

As before, consider a reasonably narrow time slice that is of size Δt. The number of reactions that is expected that occur during this time slice is

$$n_{rxn} = -\frac{dn_A(t)}{dt} \cdot \Delta t$$

This is solved to yield

$$n_{rxn} = n_A(t) 4\pi\sigma_b D \frac{n_B}{V}\left(1 + \frac{\sigma_b}{\sqrt{\pi D t}}\right)\Delta t$$

Again, these reactions are independent processes, so this is a Poisson process with the variance equal to the mean. The expected reduced reaction rate and the standard deviation are

$$E(RRR) = \frac{n_{rxn}}{n_A(t)\Delta t} = 4\pi\sigma_b D \frac{n_B}{V}\left(1 + \frac{\sigma_b}{\sqrt{\pi D t}}\right)$$

$$S.D.(RRR) = \frac{\sqrt{n_{rxn}}}{n_A(t)\Delta t}$$

These are graphed in the inset panel with solid and dashed lines, respectively. The reduced reaction rate is the number of reactions per surviving A molecule, per unit of time. It is a pseudo-first-order rate constant.

Following is the configuration file used to create the data shown in this panel:

```
/*
Bimolecular reactions, uses exact same parameters as Figure 7 of Andrews and Bray,
2004.  Lengths are in nm, times in ns.
*/

graphics none

dim 3
max_names 2
name AH
name B

max_mol 130000
rand_seed 0   # this is required to have 2000 AH molecules at time 0

difc AH 1
difc B 0

time_start -0.002
time_stop 5
time_step 0.002

boundaries 0 0 100 p
boundaries 1 0 100 p
```

```
boundaries 2 0 100 p

mol 2468 AH u u u
mol 120468 B u u u

output_files bireactABBout.txt
cmd @ 0 setrandseed -1
cmd e molcount bireactABBout.txt

reaction rxn AH + B -> 0
binding_radius rxn 0.73

end_file
```

A few aspects of this file might be surprising. The underlying reason is that the file instructs Smoldyn to place the initial AH and B molecules in the system with random locations. Some of these end up with AH and B molecules already closer than 1 binding radius, which differs from the initial condition that is assumed for the Smoluchowski equation. The solution is to add more AH and B molecules than are desired, and to start the simulation one time step before time 0. This way, the pre-existing overlaps are removed at the first time step and the simulation is in agreement with the Smoluchowski theory by time 0.

By trial and error, it was found that there would be exactly 2000 AH molecules and 120000 B molecules at time 0 if the simulation was started with 468 extra molecules of each type and used a random number seed of 0 (with Smoldyn version 1.83, which uses the system-supplied random number generator). At time 0, a runtime command is executed which randomizes the random number generator, thus yielding a different simulation result every run (although with the same starting condition).

As for panel B, we used Pearson's chi-squared statistic to see if the simulation data showed statistically significant differences from the theory. This time, 21% of the χ^2 distribution exceeded the statistic, which again showed no statistical difference between simulation and theory.

2.4 Figure 3, panel D

This panel shows the number of molecules that stick to a surface as a function of time, using various adsorption coefficients. In all cases, systems were started with a uniform density of molecules. The deterministic solution to this problem is given in Crank [2] in section 3.3.1, on the surface evaporation condition. Quoting from Crank equation 3.37, the total quantity of substance that has diffused across unit area of surface is

$$M_t = \frac{C_0 - C_2}{h}\left[e^{h^2 Dt}\operatorname{erfc}\left(h\sqrt{Dt}\right) - 1 + \frac{2}{\sqrt{\pi}}h\sqrt{Dt}\right]$$

Parameters: C_0 is the concentration that is attained at equilibrium (0 is used here because unsticking is not permitted), C_2 is the initial concentration, h is κ/D where κ is the

sticking rate constant [4] and is listed as α in Crank, and D is the diffusion coefficient. Substitution of 0 for C_0, multiplying by –1 to report molecules stuck instead of molecules gained in solution, and conversion to absolute numbers of molecules instead of concentrations leads to

$$n_{stick}(t) = \frac{n_0 A}{hV}\left[e^{h^2 Dt}\mathrm{erfc}\left(h\sqrt{Dt}\right) - 1 + \frac{2}{\sqrt{\pi}}h\sqrt{Dt}\right]$$

The new parameters are A for the surface area and V for the total system volume.

Time enters this system in two ways: through the sticking rate (κ has units of length/time) and through the diffusion coefficient (D has units of length^2/time). These parameters often occur together in the equation above, in the term h^2Dt, but not always. Thus, in contrast to our treatment of the first order reactions presented above, this situation does not lend itself to the use of a reduced time and reduced reaction rate that will allow data sets with different parameters to be compared on the same axes. Instead, fluctuations are calculated for non-reduced parameters.

As before, consider a reasonably narrow time slice of size Δt. The number of sticking events that is expected that occur during this time slice is

$$\Delta n_{stick} = \frac{dn_{stick}(t)}{dt} \cdot \Delta t$$

This is solved to yield

$$\Delta n_{stick} = \frac{n_0 AhD}{V} e^{h^2 Dt}\mathrm{erfc}\left(h\sqrt{Dt}\right)\Delta t$$

As before, this is a Poisson process. The expected sticking rate and standard deviation are

$$E(SR) = \frac{\Delta n_{stick}}{\Delta t} = \frac{n_0 AhD}{V} e^{h^2 Dt}\mathrm{erfc}\left(h\sqrt{Dt}\right)$$

$$S.D.(SR) = \frac{\sqrt{\Delta n_{stick}}}{\Delta t}$$

Following is the Smoldyn configuration file that was used to generate the simulation data that are presented in panel D:

```
# File to test molecule sticking rate

graphics opengl
graphic_iter 100

dim 3
names A B C
```

```
max_mol 50000

difc all(solution) 1

color A(solution) 1 0 0
color B(solution) 0 1 0
color C(solution) 0 0 1
color A(front) 1 0.5 0.5
color B(front) 0.5 1 0.5
color C(front) 0.5 0.5 1
display_size all 5

time_start 0
time_stop 200
time_step 0.05

boundaries 0 0 100
boundaries 1 0 100
boundaries 2 0 100

max_surface 2

start_surface
name walls
action both all reflect
color both 0 0 0
polygon both edge
max_panels rect 5
panel rect -0 100 0 0 100 100
panel rect +1 0 0 0 100 100
panel rect -1 0 100 0 100 100
panel rect +2 0 0 0 100 100
panel rect -2 0 0 100 100 100
end_surface

start_surface
name stick
rate A fsoln front 5
rate B fsoln front 0.25
rate C fsoln front 0.01
color front 1 0.7 0
color back 0.6 0 0.6
thickness 1
max_panels rect 1
panel rect +0 0 0 0 100 100
end_surface

mol 10000 A u u u
mol 10000 B u u u
mol 10000 C u u u

output_files stickrateout.txt
cmd n 2 molcountonsurf stick stickrateout.txt

end_file
```

This file was run on Smoldyn version 1.86, which uses adsorption probabilities that are correct for irreversible steady-state adsorption [5]. Prior Smoldyn versions used

equations from Erban and Chapman [4]. For molecule types A, B, and C, the respective adsorption probabilities are 0.960, 0.0938, and 0.00396, respectively.

Once again, we used Pearson's chi-squared statistic to see if the simulation data shown in the inset showed statistically significant differences from the theory. This time, 78% of the χ^2 distribution exceeded the statistic, which again showed no statistical difference between simulation and theory.

References

1. Berg HC (1993) Random Walks in Biology. Princeton, NJ: Princeton Univ. Press.
2. Crank J (1975) The Mathematics of Diffusion. Oxford: Oxford Univ. Press.
3. Andrews SS, Bray D (2004) Stochastic simulation of chemical reactions with spatial resolution and single molecule detail. Phys Biol 1: 137-151.
4. Erban R, Chapman SJ (2007) Reactive boundary conditions for stochastic simulations of reaction-diffusion processes. Phys Biol 4: 16-28.
5. Andrews SS (2009) Accurate particle-based simulation of adsorption, desorption, and partial transmission. Phys Biol 6: 46015.
