# Supplementary material for: Detailed Simulations of Cell Biology with Smoldyn 2.1
Source: PLoS Comput Biol. 2010 Mar 12;6(3):e1000705. doi: 10.1371/journal.pcbi.1000705 (PMC2837389; doi:10.1371/journal.pcbi.1000705)
Supplement: Text S3 — Runtime scales linearly with the number of molecules (0.12 MB PDF) [file pcbi.1000705.s003.pdf]

Text S3: Run time scales linearly with the number of molecules
Supplementary Information for
Detailed simulations of cell biology with Smoldyn 2.1
Andrews, Addy, Brent, and Arkin

Using various techniques, the run time of each listed simulator scales linearly with the system size [1]. Smoldyn achieves this by spatially partitioning the system volume [2,3], which does not introduce any approximations, but minimizes the number of potential interactions that need checking (see figure, below). In this method, bimolecular reactions are only considered for pairs of molecules within either the same or neighboring spatial partitions. Also, molecule-surface interactions are only considered for surface panels in the partitions that a molecule diffused through during the previous time step, assuming a straight-line trajectory. Whereas the number of chemical reactions affects run time either linearly or logarithmically in the spatial Gillespie methods [1], it has minimal effect on the run time for Smoldyn.

The following figure shows the time required to simulate a Michaelis-Menten reaction over 10^4 time steps on a MacBook Pro for a range of system sizes. The smallest system involved 10 enzyme molecules, 100 substrate molecules, and a volume of (100 nm)^3, while others were scaled up proportionately. The system was spatially partitioned for the lower points but not for the upper points. Lower and upper dashed lines illustrate linear and quadratic scaling, respectively. Parameters: the total simulated time was 10 seconds with a time step of 0.001 seconds, the association reaction rate constant was 5.9×10^4 M^{-1}s^{-1}, the dissociation reaction rate constant and product reaction rate constant were 1 s^{-1}, all molecules had diffusion coefficients of 0.01 μm^{2}s^{-1}, and where used, partitions were set up so that there would be an average of 4 molecules per partition.

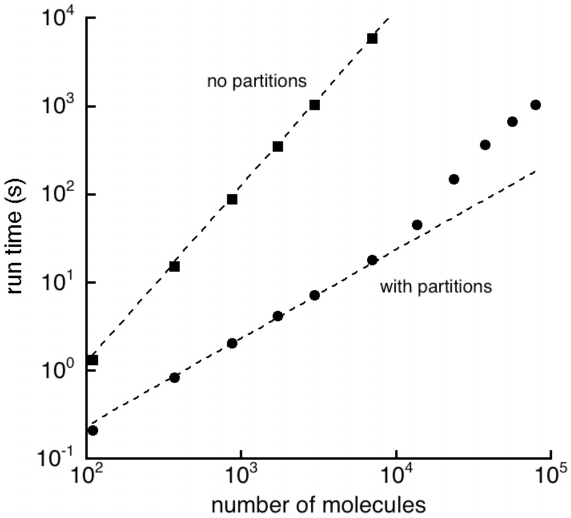

Systems with more than 10,000 molecules simulated less efficiently than smaller ones. Based on code profiling results and cross-platform comparisons, we found that this arises from computer hardware constraints: the smaller simulations kept most data in cache memory, whereas the larger ones used the slower main memory.

The configuration file for the smallest system, using partitions, is shown below. For larger systems, the boundaries were expanded and the numbers of molecules were increased so as to keep the concentrations fixed. To remove spatial partitions, one "molperbox" line was commented out, while the other was uncommented.

```
# Michaelis-Menten reaction
# units: micron and second

graphics none

dim 3
names E S ES P
max_mol 500000
#molperbox 500000
molperbox 4
accuracy 10

difc E 1
difc S 1
difc ES 1
difc P 1

color E 1 0 0
color S 0 1 0
color ES 1 1 0
color P 0 0 1

time_start 0
time_stop 10
time_step 0.001

boundaries 0 0 1 p
boundaries 1 0 1 p
boundaries 2 0 1 p

molecule_lists Elist Slist ESlist Plist
mol_list E Elist
mol_list S Slist
mol_list ES ESlist
mol_list P Plist

#output_files systemsizeout.txt
#cmd i 0 100 0.01 molcount systemsizeout.txt

# 10 E/vol and 100 S/vol
mol 10 E u u u
mol 100 S u u u

reaction fwd E + S -> ES 0.1
reaction back ES -> E + S 1
reaction prod ES -> E + P 1
product_placement back pgemmax 0.2

end_file
```

References

1. Dobrzynski M, Rodríguez JV, Kaandorp JA, Blom JG (2007) Computational methods for diffusion-influenced biochemical reactions. Bioinformatics 23: 1967-1977.
2. Andrews SS, Bray D (2004) Stochastic simulation of chemical reactions with spatial resolution and single molecule detail. Phys Biol 1: 137-151.
3. Stiles JR, Bartol TM (2001) Monte Carlo methods for simulating realistic synaptic microphysiology using MCell. In: De Schutter E, editor. Computational Neuroscience: Realistic Modeling for Experimentalists. Boca Raton, FL: CRC Press. pp. 87-130.
