# Supplementary material for: Detailed Simulations of Cell Biology with Smoldyn 2.1
Source: PLoS Comput Biol. 2010 Mar 12;6(3):e1000705. doi: 10.1371/journal.pcbi.1000705 (PMC2837389; doi:10.1371/journal.pcbi.1000705)
Supplement: Text S4 — Bar1 model details (0.15 MB PDF) [file pcbi.1000705.s004.pdf]

<u>**Text S4: Bar1 model details**</u>

Supplementary Information for

Detailed simulations of cell biology with Smoldyn 2.1

Andrews, Addy, Brent, and Arkin

4.1 Configuration files

We performed our simulations for the Bar1 example system with the following Smoldyn configuration file. We used this file for the "Bar1^{+}" data points, and commented out the "`define WITHBAR1`" line for the "Bar1^{-}" data points.

```
# Bar1 Smoldyn simulation
# by Steve Andrews, 11/30/09
# Ref: Andrews, Addy, Brent, Arkin, "Detailed simulations of cell biology with
Smoldyn 2.1", PLoS Comp. Biol. 2010
# This file is Bar29.txt, from Bar28.txt
# This is Bar1+, 1 target cell, no Bar1 adsorption
# Units: microns and seconds

define WITHBAR1

# *** output file ***
define OUTFILE1      FILEROOTout.txt
define OUTFILE2      FILEROOTout2.txt

# *** time ***
define TIMEEND 4500

# *** boundaries ***
define XLO    -12
define XHI    12
define YLO    -12
define YHI    12
define ZLO    -12
define ZHI    12

# *** model parameters ***
define NGPCR  6622   # number of GPCR
define K1t    250    # target alpha production rate
define K1c    12.5   # challenger alpha production rate
define K4     100    # Bar1 production rate in um^-2/s
define K5     5.15   # Bar1-alpha reaction rate, diff. limit is 10.3 um^3/s
define K6     0.008303     # alpha binding to GPCR, *** 2x
define K7     0.02   # alpha unbinding from GPCR, *** 2x

graphics opengl
graphic_iter 10000
frame_thickness 0
accuracy 10

dim 3
```

```
species Bar1 alpha GPCR GPCRalpha
max_mol 300000
boxsize 0.5

molecule_lists list3 list4 list1 list2 list5

mol_list alpha(fsoln) list1
mol_list alpha(up) list2
mol_list GPCR(up) list3
mol_list GPCRalpha(up) list4
mol_list Bar1(fsoln) list5
mol_list Bar1(up) list2

difc Bar1(fsoln) 27
difc alpha(fsoln) 132

color Bar1(fsoln) 0 1 0
color Bar1(front) 0 1 0
color alpha(fsoln) 0 0 0
color GPCR(up) 0 0 1
color GPCRalpha(up) 1 0 0.2

display_size alpha(fsoln) 1
display_size alpha(up) 0
display_size Bar1(all) 1
display_size GPCR(up) 2
display_size GPCRalpha(up) 2

time_start 0
time_stop TIMEEND
time_step 0.02

boundaries 0 XLO XHI
boundaries 1 YLO YHI
boundaries 2 ZLO ZHI

max_surface 5

start_surface sides
polygon both none
unbounded_emitter front Bar1 K4 0 0 0
unbounded_emitter front alpha K1t 5.5 0 0
unbounded_emitter front alpha K1c 2.75 4.7632 0
unbounded_emitter front alpha K1c -2.75 4.7632 0
unbounded_emitter front alpha K1c -5.5 0 0
unbounded_emitter front alpha K1c -2.75 -4.7632 0
unbounded_emitter front alpha K1c 2.75 -4.7632 0
read_file ellipse_12_12.txt
end_surface

start_surface cell
action both all reflect
polygon both face
```

```
color both 0.8 0.8 0.8
max_panels sphere 1
panel sphere 0 0 0 2.5 20 20
end_surface

start_surface alphatarget
polygon both face
color both 0.3 0.3 0.3
max_panels sphere 6
panel sphere 5.5    0      0 2.5 20 20
end_surface

start_surface alphachallenge
polygon both face
color both 0.5 0.5 0.5
max_panels sphere 6
panel sphere 2.75   4.7632 0 2.5 20 20
panel sphere -2.75  4.7632 0 2.5 20 20
panel sphere -5.5   0      0 2.5 20 20
panel sphere -2.75  -4.7632      0 2.5 20 20
panel sphere 2.75   -4.7632      0 2.5 20 20
end_surface

surface_mol NGPCR GPCR(up) cell all all

reaction_surface alphatarget rxn1t 0 -> alpha(fsoln) K1t    # alpha production
reaction_surface alphachallenge rxn1c 0 -> alpha(fsoln) K1c # alpha production

ifdefine WITHBAR1
  reaction_surface cell rxn4 0 -> Bar1(fsoln) K4            # Bar1 production
  reaction rxn5 Bar1(fsoln) + alpha(fsoln) -> Bar1(fsoln) K5 # Bar1 protease
endif

reaction rxn6 GPCR(up) + alpha(fsoln) -> GPCRalpha(up) K6   # GPCR-alpha on
reaction rxn7 GPCRalpha(up) -> GPCR(up) + alpha(fsoln) K7    # GPCR-alpha off
product_placement rxn7 pgemmax 0.2

cmd @ 0 set reaction_rate rxn1t 15.625
cmd @ 500 set reaction_rate rxn1t 31.25
cmd @ 1000 set reaction_rate rxn1t 62.5
cmd @ 1500 set reaction_rate rxn1t 125
cmd @ 2000 set reaction_rate rxn1t 250
cmd @ 2500 set reaction_rate rxn1t 500
cmd @ 3000 set reaction_rate rxn1t 1000
cmd @ 3500 set reaction_rate rxn1t 2000
cmd @ 4000 set reaction_rate rxn1t 4000

cmd @ 0 set reaction_rate rxn1c 0.78125
cmd @ 500 set reaction_rate rxn1c 1.5625
cmd @ 1000 set reaction_rate rxn1c 3.125
cmd @ 1500 set reaction_rate rxn1c 6.25
cmd @ 2000 set reaction_rate rxn1c 12.5
cmd @ 2500 set reaction_rate rxn1c 25
```

```
cmd @ 3000 set reaction_rate rxn1c 50.0
cmd @ 3500 set reaction_rate rxn1c 100.0
cmd @ 4000 set reaction_rate rxn1c 200.0

output_files stdout OUTFILE1 OUTFILE2

cmd B molcountheader stdout
cmd B molcountheader OUTFILE2
cmd i 0 TIMEEND 2 molcount stdout
cmd i 0 TIMEEND 2 molcount OUTFILE2
cmd i 0 TIMEEND 2 molmoments GPCRalpha(up) OUTFILE1

end_file
```

In the code for the surface called "sides", this configuration file refers to the file ellipse_12_12.txt. That is a simple list of 480 triangles that approximate the surface of a sphere which has a radius of 12 μm. We generated these data by generating a unit sphere in Mathematica, triangulating it with Mathematica's TriangularSurfacePlot function, and storing the result in the Virtual Reality Modeling Language (VRML) format. Then, we converted the data to Smoldyn format, oriented the triangles, and scaled the sphere to a radius of 12 μm using the wrl2smol utility program. Following is an excerpt of ellipse_12_12.txt:

```
# Smoldyn surface data file automatically generated by wrl2smol
# input file: ellipsetop.wrl ellipsebot.wrl
# output file: ellipse_12_12.txt

max_panels tri 480

panel tri  0 0 12  2.16288 0.895894 11.7694  2.34108 0 11.7694  tri1
panel tri  0 0 12  2.34108 0 11.7694  2.16288 -0.895894 11.7694  tri2
panel tri  0 0 12  1.6554 1.6554 11.7694  2.16288 0.895894 11.7694  tri3
...
panel tri  1.6554 -1.6554 -11.7694  2.16288 -0.895894 -11.7694  0 0 -12  tri480

end_file
```

4.2 Model parameters

System boundary. The simulated system was bounded by a nearly spherical surface, composed of 480 triangles, that had a radius of 12 μm and was centered about the *MAT***a** cell.

Tests with systems that were up to 37 times larger in volume indicated that the system sizes used here were fully sufficient for $Bar1^-$ simulations but caused too few α-factor proteolysis reactions to occur for $Bar1^+$ simulations. This, in turn, caused the simulated populations of α-factor-GPCR complexes to be between 5 and 10% too large, in $Bar1^+$ simulations. As a result, the EC_{50} shift shown in Figure 4B, which is a factor of 5.2, is between 5 and 10% too small, compared to how it would be with unbounded

systems. Errors for other portions of Figure 4B are likely to be similar. This has a small effect on our quantitative results and does not affect any of our qualitative conclusions.

This boundary absorbed α-factor and Bar1 using absorption coefficients that emulated an unbounded system reasonably well. Smoldyn calculated these coefficients separately for α-factor and Bar1, and separately for each bounding triangle. This "unbounded-emitter" method is mentioned in the main text and described in detail in [1].

System geometry. We modeled each yeast cell as a 5 μm diameter sphere. The *MAT***a** cell was at the origin and the *MAT*α cells surrounded it, as Figure 4A shows. Cells were spaced 0.5 μm apart from each other.

Simulation time step. We chose a simulation time step of 0.02 s. This is relatively long time step, but we determined that it was adequate using preliminary simulations which showed minimal changes of results between time steps of 0.01 s and 0.05 s. Using relatively long steps substantially decreased the computational intensity of simulations.

Diffusion coefficients. We calculated α-factor and Bar1 diffusion coefficients using the Stokes-Einstein equation, which is

$$D = \frac{k_B T}{6\pi\eta\sigma}$$

where D is the diffusion coefficient, k_B is Boltzmann's constant, T is the temperature, η is the solution viscosity, and σ is the particle radius. We assumed a temperature of 37°C. We assumed a viscosity of 2 Pa s, which is roughly the viscosity for a mammalian cell cytoplasm [2], because this seemed like a reasonable estimate for the viscosity within a yeast ascus or other natural yeast environments. We estimated the α-factor molecular radius using its molecular weight of 1.684 kDa and a density equal to that of water; from this, we calculated its diffusion coefficient to be about 132 $\mu m^2/s$. The Bar1 molecular weight is more complicated, since the protein weight is 64 kDa [3], but it is reported to be heavily glycosylated to a weight of more than 200 kDa [4]. We chose a weight of 200 kDa and again a density equal to that of water, which led to a diffusion coefficient of 27 $\mu m^2/s$.

With the simulation time step quoted above, 0.02 s, the rms step lengths for α-factor and Bar1 were 2.30 μm and 1.04 μm, respectively. These are very long rms step lengths, but are still sufficiently less than the cell diameters to minimally affect spatial concentration distributions. Preliminary simulations showed that concentration profiles were essentially the same with time steps between 0.01 s and 0.05 s.

α-factor secretion rate. Simulations started with the target cell α-factor production rate equal to 15.625 $\mu m^{-2}s^{-1}$ (which is multiplied by the *MAT*α cell surface area of 78.5 μm^2 to yield a total cell secretion rate of 1230 s^{-1}). Challenger cell α-factor production rates were 5% of the target cell rate. After every 500 s of simulated time, these production rates were doubled. Maximum production rates were 4000 $\mu m^{-2}s^{-1}$ for the

target cell (314,000 s^{-1} for the whole cell) and 200 $\mu m^{-2}s^{-1}$ for the challenger cells. The abscissa for Figure 4B represents the target cell release rate, considering the whole cell surface. We used this value for the abscissa because it is unambiguous. In contrast, the α-factor concentration at the *MAT***a** cell is ambiguous because it depends on the presence of Bar1 and it has a strong spatial gradient. If desired, a rough correspondence can be established using the fact that the simulated EC_{50} for receptor binding was 4.7 nM α-factor (see below) and the simulated EC_{50} for α-factor secretion and a $Bar1^-$ *MAT***a** cell was 260 α-factor molecules $\mu m^{-2}s^{-1}$ (20,400 s^{-1} for the whole cell)

The target cell released an average of 25 molecules per time step for the smallest release rate and 6280 molecules per time step for the largest release rate.

Bar1 secretion rate. Our simulated Bar1 production rate was 100 $\mu m^{-2}s^{-1}$ (which is multiplied by the *MAT***a** cell surface area of 78.5 μm^2 to yield a total cell secretion rate of 7850 s^{-1}). We chose this value because, combined with the proteolysis reaction rate (see below), it yielded about a 5-fold shift in the EC_{50} for pheromone binding. We chose this 5-fold shift because it is large enough to show a strong effect, but is still small compared with shifts that arise in several mutant strains [5,6]; experimental results are not available for the actual quantitative effect of Bar1 on pheromone response.

The *MAT***a** cell released an average of 157 Bar1 molecules each time step and the entire system volume contained about 18,600 Bar1 molecules at any time.

Bar1-α proteolysis reaction rate. We set the reaction rate constant for the proteolysis of α-factor by Bar1 to 5.15 $\mu m^3/s$, which is $3.1 \times 10^9\ M^{-1}s^{-1}$. This is an unphysically high reaction rate. We intentionally chose a high value though because it meant that we could achieve a 5-fold shift in the EC_{50} for pheromone binding, as described above, while simulating relatively few Bar1 molecules, and thus achieving relatively fast simulations. Because Bar1 molecules were still present at a concentration of about 2.6 μm^{-3} on average, and concentrations were much higher close to the *MAT***a** cell, our relatively low Bar1 concentrations were unlikely to have caused significant spatial correlations between reactions.

The binding radius for this reaction, using our time step of 0.02 seconds, was 0.291 μm. This is clearly much larger than the sum of the physical Bar1 and α-factor radii. However, it is still much smaller than the cell radii (2.5 μm), so we expect that any reaction artifacts near cell surfaces were minimal. On the other hand, we were concerned that these large binding radii might cause individual Bar1 molecules to be within a binding radius of multiple α-factor molecules on a regular basis, especially with high α-factor release rates. This would be a problem because each Bar1 can only react once per time step, thus causing effective saturation of Bar1. This was in fact a problem when we used a proteolysis reaction rate of 10.3 $\mu m^3/s$, which is the diffusion-limited reaction rate for the model system. We found that it was a problem because the GPCR-α-factor binding dose-response curve did not fit a Hill function, as it should for a simple binding system, but instead showed too high GPCR-α-factor binding at high α-factor doses. For this reason, we halved the reaction rate to 5.15 $\mu m^3/s$; using this rate, Hill functions fit dose-response curves essentially perfectly (the Hill coefficient for the $Bar1^+$ dose-

response curve shown in Figure 4B is 0.98), which indicates that any Bar1 saturation is negligible.

α-factor binding to GPCR. Yi and coworkers reported that the α-factor-GPCR association and dissociation rate constants are 2×10^6 $M^{-1}s^{-1}$ (0.00332 $\mu m^3 s^{-1}$) and 0.01 s^{-1}, respectively [5]. These imply a dissociation constant of 5 nM. For consistency with other work [7], we aimed to use a dissociation constant of 4 nM, which is well supported by experimental results [8,9]. For this reason, we assumed a slightly larger association rate constant of 0.00415 $\mu m^3 s^{-1}$, and kept Yi et al.'s dissociation rate of 0.01 s^{-1}. These values are in good agreement with experimental binding rates, which Jenness and coworkers measured [9,10].

These on- and off-rates are sufficiently slow that equilibration between receptor-ligand complexes and the solution (~100 s half-life) is much slower than other simulation timescales (*e.g.* a Bar1 molecule diffuses across the entire simulation volume in about 10 s). As a result, the model is a "stiff" system, with many fast timescale processes and the single slow ligation process that determines the overall equilibration rate. To speed up the simulations, we doubled both the on- and off-rates for α-factor binding to GPCR, to 0.00830 $\mu m^3 s^{-1}$ and 0.02 s^{-1}, respectively. This caused our simulated ligation dynamics to be a factor of 2 too fast, but it did not affect the equilibrium properties, including the results presented in Figure 4.

The association binding radius was 0.043 μm. By comparison, the *MAT***a** cell surface area was 78.5 μm^2 and it was covered with 6622 randomly positioned GPCR receptors [11], so the average spacing between receptors was 0.11 μm. The binding radius was sufficiently smaller than the average spacing between receptors that relatively few receptors had binding regions that overlapped those of their neighbors, thus minimizing interactions that could have caused artifacts. Nevertheless, some receptor binding regions did overlap those of their neighbors. For this reason, the simulated dissociation constant was not quite equal to the 4 nM that we aimed for, but was actually 4.7 nM.

Simulation performance

The $Bar1^-$ simulation took 13 hours to run and the $Bar1^+$ simulation took 8 hours (the latter was faster because it had fewer total molecules, due to degradation of α-factor by Bar1), using a 2009 Mac Pro computer, running OS 10.6 and Smoldyn 2.09. Preliminary simulations, from which we were able to find all of the qualitative results shown in Figure 4 but which lacked quantitative accuracy, took about 30 minutes each.

References

1. Andrews SS (2009) Accurate particle-based simulation of adsorption, desorption, and partial transmission. Phys Biol 6: 46015.

2. Mastro AM, Babich MA, Taylor WD, Keith AD (1984) Diffusion of a small molecule in the cytoplasm of mammalian cells. Proc Natl Acad Sci USA 81: 3414-3418.
3. Bardwell L (2005) A walk-through of the yeast mating pheromone response pathway. Peptides 26: 339-350.
4. Ballensiefen W, Schmitt HD (1997) Periplasmic Bar1 protease of *Saccharomyces cerevisiae* is active before reaching its extracellular destination. Eur J Biochem 247: 142-147.
5. Yi T-M, Kitano H, Simon MI (2003) A quantitative characterization of the yeast heterotrimeric G protein cycle. Proc Natl Acad Sci USA 100: 10764-10769.
6. Yu RC, Pesce CG, Colman-Lerner A, Lok L, Pincus D, et al. (2008) Negative feedback that improves information transmission in yeast signalling. Nature 456: 755-761.
7. Andrews SS, Yu R, Brent R (2009) Cell signaling dose response alignment may arise from cooperativity or pull-up/pull-down mechanisms. In preparation.
8. Bajaj A, Celic A, Ding F-X, Naider F, Becker JM, et al. (2004) A fluorescent alpha-factor analogue exhibits multiple steps on binding to its G protein coupled receptor in yeast. Biochem 43: 13564-13578.
9. Jenness DD, Burkholder AC, Hartwell LH (1986) Binding of alpha-factor pheromone to *Saccharomyces cerevisiae* **a** cells: dissociation constant and number of binding sites. Mol Cell Biol 6: 318-320.
10. Jenness DD, Burkholder AC, Hartwell LH (1983) Binding of alpha-factor pheromone to yeast a cells: chemical and genetic evidence for an alpha-factor receptor. Cell 35: 521-529.
11. Thomson TM, Benjamin KR, Bush A, Love T, Yu RC, et al. (2009) Scaffold number in yeast signaling system sets tradeoff between system output and dynamic range. In preparation.
